# Translocator protein (18kDA) (TSPO) marks mesenchymal glioblastoma cell populations characterized by elevated numbers of tumor-associated macrophages

**DOI:** 10.1186/s40478-023-01651-5

**Published:** 2023-09-11

**Authors:** Lorraine Weidner, Julia Lorenz, Stefanie Quach, Frank K. Braun, Tanja Rothhammer-Hampl, Laura-Marie Ammer, Arabel Vollmann-Zwerenz, Laura M. Bartos, Franziska J. Dekorsy, Adrien Holzgreve, Sabrina V. Kirchleitner, Niklas Thon, Tobias Greve, Viktoria Ruf, Jochen Herms, Stefanie Bader, Vladimir M. Milenkovic, Louisa von Baumgarten, Ayse N. Menevse, Abir Hussein, Julian Sax, Christian H. Wetzel, Rainer Rupprecht, Martin Proescholdt, Nils O. Schmidt, Philipp Beckhove, Peter Hau, Joerg-Christian Tonn, Peter Bartenstein, Matthias Brendel, Nathalie L. Albert, Markus J. Riemenschneider

**Affiliations:** 1 Department of Neuropathology, Regensburg University Hospital, Franz-Josef-Strauß-Allee 11, 93053 Regensburg, Germany; 2 Wilhelm Sander Neuro-Oncology Unit, Regensburg University Hospital, Regensburg, Germany; 3 Department of Neurosurgery, University Hospital of Munich, LMU Munich, Munich, Germany; 4 Department of Neurology, Regensburg University Hospital, Regensburg, Germany; 5 Department of Nuclear Medicine, University Hospital of Munich, LMU Munich, Munich, Germany; 6 Center for Neuropathology and Prion Research, LMU Munich, Munich, Germany; 7 Department of Psychiatry and Psychotherapy, University Regensburg, Regensburg, Germany; 8 Division of Interventional Immunology, Leibniz Institute for Immunotherapy, Regensburg, Germany; 9 Department of Neurosurgery, University Hospital Regensburg, 93053 Regensburg, Germany; 10 Department of Internal Medicine III, University Hospital Regensburg, Regensburg, Germany; 11 German Center for Neurodegenerative Diseases (DZNE) and Munich Cluster for Systems Neurology (SyNergy), Munich, Germany

**Keywords:** TSPO, Glioma, PET, Imaging, Promotor methylation, RNA seq, Immunohistochemistry, Intratumoral heterogeneity, Microglia, Myeloid cells

## Abstract

TSPO is a promising novel tracer target for positron-emission tomography (PET) imaging of brain tumors. However, due to the heterogeneity of cell populations that contribute to the TSPO-PET signal, imaging interpretation may be challenging. We therefore evaluated TSPO enrichment/expression in connection with its underlying histopathological and molecular features in gliomas. We analyzed *TSPO* expression and its regulatory mechanisms in large *in silico* datasets and by performing direct bisulfite sequencing of the *TSPO* promotor. In glioblastoma tissue samples of our TSPO-PET imaging study cohort, we dissected the association of TSPO tracer enrichment and protein labeling with the expression of cell lineage markers by immunohistochemistry and fluorescence multiplex stains. Furthermore, we identified relevant TSPO-associated signaling pathways by RNA sequencing.

We found that *TSPO* expression is associated with prognostically unfavorable glioma phenotypes and that *TSPO* promotor hypermethylation is linked to *IDH* mutation. Careful histological analysis revealed that TSPO immunohistochemistry correlates with the TSPO-PET signal and that TSPO is expressed by diverse cell populations. While tumor core areas are the major contributor to the overall TSPO signal, TSPO signals in the tumor rim are mainly driven by CD68-positive microglia/macrophages. Molecularly, high *TSPO* expression marks prognostically unfavorable glioblastoma cell subpopulations characterized by an enrichment of mesenchymal gene sets and higher amounts of tumor-associated macrophages.

In conclusion, our study improves the understanding of TSPO as an imaging marker in gliomas by unveiling IDH-dependent differences in TSPO expression/regulation, regional heterogeneity of the TSPO PET signal and functional implications of TSPO in terms of tumor immune cell interactions.

**Supplementary Information:**

The online version contains supplementary material available at 10.1186/s40478-023-01651-5.

## Introduction

Adult-type diffuse gliomas are the most frequent malignant brain tumors [54] and diagnosed by histological and molecular features according to the 2021 World Health Organization (WHO) classification of tumors of the central nervous system. They comprise the entities of oligodendroglioma, IDH-mutant and 1p/19q-codeleted (WHO grades 2–3), astrocytoma, IDH-mutant (WHO grade 2–4) and glioblastoma, IDH-wildtype (WHO grade 4) [45]. Prognosis differs between gliomas: While IDH-mutant gliomas have a more favorable prognosis, survival times for most glioblastoma (GBM) patients range between 15 and 18 months [54].

Unsupervised clustering of methylation array profiles defines distinct molecular subtypes of adult-type diffuse gliomas with IDH-mutant gliomas clustering closely together and clearly separating from IDH-wildtype GBMs [45]. *IDH* mutations are commonly associated with a genome-wide hypermethylation phenotype [16], while molecular heterogeneity within IDH-wildtype GBMs is described by transcriptome-wide RNA sequencing profiles [69]. Currently there are three clinically relevant GBM subgroups referred to as proneural (PN), mesenchymal (MES) and classical (CL) [9, 12, 16, 34, 56, 72]. Recently, single cell RNA sequencing revealed four main cellular states in GBMs: neural progenitor-like (NPC1/2-like), oligodendrocyte progenitor-like (OPC-like), astrocyte-like (AC-like) and mesenchymal-like (MES1/2-like). MES-like cells are more abundant in the MES subgroup, are linked to tissue microenvironment interaction [52] and are promoted by macrophage-derived Oncostatin M (OSM) that interacts with its receptors (OSMR and LIFR) in complex with GP130 via STAT3 signaling pathways [30]. Imaging markers to identify molecular subgroups and cellular composition are of great need, especially to support improved precision immunotherapy treatment approaches, like neoadjuvant anti-PD-1 therapy in case of high cell proportions in the tumor microenvironment (TME) or different options targeting tumor-associated macrophages (TAMs) [19, 74].

TSPO is a transmembrane protein located in the outer mitochondrial barrier. The gene is located at 22q13.31 and contains four exons [24]. It has been associated with a broad spectrum of functions such as steroid synthesis, regulation of proliferation, apoptosis and migration, as well as mitochondrial functions such as mitochondrial respiration and oxidative stress regulation [3]. Its expression is regulated by a GC-rich promotor in breast cancer cell lines that contains binding sites for several transcription factors, including SP1 and SP3 [7]. Evidence exists that the PKCε-ERK1/2-AP1-STAT3 signaling pathway can initiate *TSPO* transcription by upregulation of ETS and SP1/SP3 transcription factors (TFs) in MA-10 Leydig cells [6]. However, *TSPO* transcriptional regulation in gliomas is still poorly understood. According to GTEx v8 (GTEx data release 8, dbGaP Accession phs000424.v8.p2, accessed 19.12.2021), *TSPO* is normally expressed at very low levels in the central nervous system (CNS) compared to other healthy tissues [1, 26]. However, its expression is upregulated at sites of inflammation or neurodegeneration [53] and also in gliomas [14, 71, 79]. TSPO-PET imaging is a potential prognostic marker in patients suffering from diffuse gliomas [14, 58]. It has been described to mark the tumor microenvironment (TME) with its myeloid compartment and to indicate therapy-induced changes during tumor progression [22, 57, 79]. Regarding the cellular source of TSPO-PET, tumor cells, reactive astrocytes, endothelial cells, and macrophages/ microglia have been discussed [33, 53, 60, 79]. This suggests that the presence of different cell populations and their interplay might generate a cumulative TSPO signal depending on the imaging sampling area and time point. However, further histopathological approaches to better understand TSPO imaging correlates are urgently needed.

In this study, we investigate the histological and molecular correlates of TSPO-PET tracer uptake and TSPO tissue expression. We used *in silico* data to get an overview of *TSPO* expression in low- and high-grade gliomas. Furthermore, we analyzed open-access data to assess *TSPO* methylation, mutation and amplification as potential regulatory mechanisms of *TSPO* expression. With results hinting to *TSPO* gene silencing by promotor hypermethylation, we performed direct bisulfite sequencing and qPCR analyses on an own tumor cohort. Most importantly, we used tissues from our unique TSPO-PET imaging study cohort that underwent TSPO-PET imaging and targeted biopsy or resection allowing for the direct correlation between tracer enrichment and histopathological/molecular features. In this cohort, we studied the regional and cellular heterogeneity of TSPO expression and used RNA sequencing (RNA-Seq) to elucidate *TSPO* signaling relationships.

## Materials and methods

### Patient samples and tissue specimens

Fresh-frozen tumor tissues for bisulfite PCR methylation analysis and antibody validation were selected from the tumor tissue archive of the Department of Neuropathology, Regensburg University Hospital and investigated according to protocols approved by the institutional review board (ethics board approval no. 18-1207-101 and 20-1799-101). Tumors were initially classified according to the WHO 2016 and re-classified according to the WHO 2021 classification [45]. Parts of each tumor were snap-frozen directly after surgical resection and stored at -80 C. Only tissue samples with a tumor cell content of 70% or more were used for methylation analyses (Suppl. Table 1). 2 non-neoplastic brain samples from different individuals (NB01, NB02) served as a reference (D1234062, Biochain and X11001-1, Epigentek). As further controls, we employed commercially available hypermethylated DNA (S7821; Millipore) and unmethylated blood DNA. Antibody validation was performed on protein lysates of 4 IDH-wildtype glioblastomas (GBMs), WHO grade 4.

We used 26 IDH-wildtype GBM (biopsies/resections) from our FOR2858 (German Research Foundation, DFG) TSPO-PET imaging study (Suppl. Table 2) [58], collected in the Center for Neurosurgery/Neuropathology/Department of Nuclear Medicine, University Hospital of Munich (LMU Munich, Munich Germany) in line with local ethics board approval (ethics board approval no. 18–783). All patients had received contrast-enhanced MRI, TSPO-PET and amino acid PET within a maximum of 18 and a median of 3 days before the operation. MRI included gadolinium-enhanced T1- (1 mm slices) and T2‐weighted scans (2 mm slices). For TSPO-PET, approximately 180 MBq [^18^F]GE180 were injected intravenously. Summation scans 60 to 80 min post injection were used for image analysis. For amino acid PET, approximately 180 MBq [^18^F]FET were injected and 40 min post injection summation images were analyzed as described previously [68]. PET scans were performed on 2 subsequent days. Due to the short half-life of F18 (110 min) there was no signaling overlap. Areas of interest were defined in an interdisciplinary exchange between the attending neurosurgeon and nuclear medicine specialist. Brainlab planning software (Brainlab) was used for image fusion and either biopsy planning (Suppl. Figure 1), or intraoperative navigation in case of open tumor resection. [^18^F]GE180 and FET uptake at the exact localization of the acquired tissue specimen were retrospectively measured by fusing the intraoperative CT or intraoperatively acquired navigation points with the PET images using a Hermes workstation (Hermes Medical Solutions). Standard histological and molecular assessment for diagnostic purposes was performed at the Center for Neuropathology and Prion Research LMU Munich according to WHO criteria as described above. Formalin-fixed and paraffin-embedded (FFPE) tissues from all 26 patients with IDH-wildtype GBM (18 newly diagnosed and 8 recurrent tumors) were used for further immunohistochemical analyses in Regensburg and assessment of tumor cell content within the framework of this study. The tumor cell content of each specimen was evaluated histologically at the Department of Neuropathology at Regensburg University Hospital using H&E stains and classified into one of four categories by the following histologic criteria: “no tumor”, characterized by cortex and white matter with no visible tumor cells; “some tumor”, characterized by cortex and satellitoses or only sporadic infiltrating tumor cells; “infiltration zone”, characterized by a low tumor cell content intermixed with non-neoplastic tissue; “solid tumor”, characterized by a tumor cell content of at least 70–80%. For whole transcriptome analyses (bulk RNA-Seq), fresh-frozen tumor tissue from a subset of 18 patients with IDH-wildtype GBM was available (Suppl. Table 3).

### *In silico* data sets and cell lines

Open-access HTSeq count data from The Cancer Genome Atlas (TCGA) database were used (TCGA-LGG/-GBM project: n = 630 quality-controlled cases, 38.3% female, age mean = 47.3 ± 15.23, access: 28.02.2021). WHO grade according to WHO 2016 [44] and *IDH* mutational status was reported for n = 566 cases: 36.40% had WHO grade II glioma (18 IDH-wildtype, 188 IDH-mutant), 40.11% had WHO grade III (66 IDH-wildtype, 161 IDH-mutant), and 23.50% had WHO grade IV glioma (127 IDH-wildtype, 6 IDH-mutant). Regarding expression subtypes, 56 IDH-wildtype GBM were mesenchymal, 43 IDH-wildtype GBM were classical, and 12 IDH-wildtype plus 4 IDH-mutant GBM were proneural.

Open-access K450 Illumina methylation array data from the TCGA Research Network [51] were used (access: 01.04.2020, 04.11.2020) covering the *TSPO* CpG island locus (22q13.2, chr22: 43,151,314–43,152,163, 850 bp length). After excluding cases with uncertain annotations, 130 high-grade (TCGA-GBM) and 515 low-grade gliomas (TCGA-LGG) were left for evaluation. Entities analyzed included 76 anaplastic oligodendrogliomas (AOD, ICD-O code: 9451/3), 118 not otherwise specified oligodendrogliomas (NOS OD, ICD-O code: 9450/3), 130 mixed gliomas (MG, ICD-O code: 9382/3), 62 not otherwise specified astrocytomas (NOS A, ICD-O code: 9400/3), 129 anaplastic astrocytomas (AA, ICD-O code: 9401/3), and 130 glioblastomas (GBM, ICD-O code: 9440/3).

U251MG and U87MG GBM cells were obtained for antibody validation from Cell Lines Service GmbH (Eppelheim, Germany) and CRISPR/Cas9 TSPO-knockout microglia cells were provided by Prof. Dr. Christian Wetzel, Regensburg [49].

### *In silico* analyses

For *TSPO* mRNA expression analyses, open-access HTSeq count data from the TCGA database were used (TCGA Research Network, https://www.cancer.gov/tcga). We used data.table (v1.14.2, [21]) and R.utils (v2.12.2, [8]) R packages to decompress and extract counts. We linked IDs with the provided genecode v22 reference and calculated gene expression values (reads per kilobase per million, RPKM/ for dREG deconvolution: transcripts per million, TPM) for each gene and case ID. We extracted high-grade GBM (IDH-wildtype/-mutant) and low-grade astrocytoma (IDH-wildtype/-mutant) cases based on their histology information published in [16]. GBM expression subtypes and GBM methylation subtypes were also annotated from that source.

For analyses of promotor methylation at the *TSPO* CpG island locus, open-access K450 methylation array data generated by TCGA Research Network were used. Data.table (v1.14.2, [21]) and tidyverse (v1.3.2, [75]) R packages were used to extract beta values for chromosome 22 and filtered for TSPO gene area (n = 15 probes) and CpG island area (n = 7 probes). Utilizing additional data from TCGA, we linked IDs and clinical information for each patient to the array probe data (CpG island probes: cg00343092, cg01633858, cg08314021, cg10822314, cg20390150, cg24899361, cg26131049).

We utilized low-grade and high-grade glioma datasets using UALCAN and cBioportal queries for analyzing *ETS1* and *ETS2* expression patterns.

### Bisulfite PCR methylation analysis

We performed bisulfite PCR methylation analysis for 72 of the 80 *TSPO* CpG island-related CpGs from fresh-frozen tumor tissues (Suppl. Table 1). DNA was isolated according to the manufacturer’s instructions using AllPrep DNA/RNA Mini kit (#80,204, Qiagen). For bisulfite conversion of DNAs we used the EZ DNA Methylation-Gold™ kit (D5005, Zymo Research Europe GmbH). Methylation-specific oligonucleotides were designed using bisulfite primer seeker (Zymo Research, available under: https://www.zymoresearch.de/pages/bisulfite-primer-seeker). Oligonucleotide sequences and PCR conditions used are listed in Suppl. Table 4. PCR products were sequenced at StarSEQ laboratory (Mainz, Germany). The resulting sequences were evaluated with Chromas software v2.6.6 (Technelysium) as described previously [61]. TF binding site predictions were calculated for the whole *TSPO* CpG island region (850 bp) with JASPAR 9th release of 2022 [15].

### Quantitative real-time reverse transcription PCR analysis for *TSPO* transcript quantification

RNA was isolated using the AllPrep DNA/RNA Mini kit from Qiagen. cDNA synthesis was performed with random hexamer primers (#26-4000-03, Gene Link) and the SuperScript™ II Reverse Transcriptase kit (#18064-022, Invitrogen) according to the manufacturer’s instructions. Quantitative real-time (reverse transcription) PCR (qRT-PCR) was performed with SensiFAST™ SYBR Hi-Rox kit (BIO-92,005, Bioline) on a StepOnePlus™ cycler (Life Technologies) as described [65]. Primers used for *ARF-1* control were 5´-GACCACGATCCTCTACAAGC (forward) and 5´-TCCCACACAGTGAAGCTGATG (reverse). Primers for *TSPO* detection were 5´- TCTTTGGTGCCCGACAAAT (forward) and 5´-GGTACCAGGCCACGGTAGT (reverse) and were previously described in [49].

### Immunohistochemistry, blocking experiment and scoring

IHC staining was performed using the EnVision™ Kit (K4065, Dako) as previously described [70]. Heat-induced epitope retrieval (HIER) was performed in 10 mM citrate buffer (pH 6.0, targets: TSPO/CD68). Primary antibodies were anti-TSPO (1:5000, ab109497, abcam), anti-CD68 (1:200, M0876, Dako).

Epitope blocking experiments for TSPO antibody validation were performed using human TSPO peptide (ab170987, abcam). Briefly, before primary antibody application on sections, the TSPO antibody was incubated with fivefold the amount of blocking peptide (30 min, room temperature).

IHC staining was scored using a Zeiss Imager M2 (200x, DL = 40–41%, 0.80 aperture, FL = on). Whole biopsy samples or four random scoring fields in resections were scored. The TSPO staining intensity was evaluated using a H Score-oriented approach [31]. For area analysis, %Area positive for TSPO or CD68 were estimated in at least 5 images in the case of resections and 1–3 images in the case of biopsies (400x, DL = 59.1%, 0.90 aperture, FL = on, Axiocam 503 color camera). The images were processed with Fiji Image J [64] using background subtraction (default settings), color separation, color threshold regime (moments method), binary conversion and the analyze particles module.

### Multiplex OPAL immunofluorescence staining and counting

We used the OPAL™ system (Akoya Biosciences) for staining the following target combinations: TSPO/CD68, TSPO/CD11b, TSPO/GFAP, and p53/TSPO. Briefly, paraffin sections (3 μm) were baked (50 °C), deparaffinized, and rehydrated. First target stain: HIER was performed for 30 min. After washing (PBS, 0.05% Tween® 20), sections were blocked for 10 min with kit-provided endogenous enzyme block (K4065, Dako) at room temperature (RT). Unspecific binding sites were blocked with 1% bovine serum albumin (10 min, RT). Incubation with primary antibodies was performed (1 h, RT). After washing, sections were labeled with Opal polymer HRP Ms + Rb (10 min, RT). After additional washing, sections were incubated with Opal Dye working solution (10 min, RT). Specifications for HIER buffers, primary antibodies and OPAL fluorescence dyes/work solution are provided in Suppl. Table 5. After washing again, the second target stain was performed similarly to the first one. Then, a final HIER step in 10 mM citrate buffer (pH 6.0) was followed by nuclear counterstaining using Spectral DAPI solution (1 droplet/ml TBST, pH 7.5, 0.05% Tween® 20) for 5 min at room temperature. Finally, slides were cover-slipped using Aquatex® (#1.08562.0050, Merck KGaA) and JPEG images (400x) were counted within Fiji [64] with the Cell Counter plugin [39].

### Generation of transient TSPO-knockdown protein lysates from glioma cell lines

Knockdown was achieved by transfecting glioma cells with 30nM ON-TARGETplus *TSPO* siRNA - SMARTpool (L-009559-00-0005, Dharmacon) utilizing the DharmaFECT™ system (V0318) according to manufacturer’s instructions. Transfection medium was replaced after 24 h with culture medium DMEM (10% FCS, P/S). 48 h after transfection the culture medium was removed, cells were washed, and harvested in RIPA lysis buffer containing a protease inhibitor cocktail (cOmplete ULTRA Tablets, mini, EDTA-free, Roche). Protein lysates were mixed (15 min, 4 °C) and centrifuged (10 min, 4 °C, 4754 g). Using a modified Lowry approach, the protein content was measured using a Bio-Rad DC™ protein assay (#5,000,112, Bio-Rad) on a FLUOstar Omega reader (BMG Labtech).

### Immunoblotting and blocking experiment for antibody validation

We performed western blots with transient TSPO-knockdown glioma cell lines and glioblastoma protein lysates according to [65] with following specifications: Primary antibody was anti-TSPO antibody (1:10 000; ab109497 [EPR 5384], abcam) in 5% milk/TBST (0.1% Tween® 20). In case of an epitope blocking experiment, the primary antibody was incubated with fivefold the amount of human TSPO peptide (ab170987, abcam) for 30 min at room temperature prior to its application on the membrane. The secondary antibody was goat-anti-rabbit linked to HRP (1:10 000; #31,460, Thermo Scientific) for 60 min at room temperature. Signal visualization was accomplished with SuperSignal™ kit (#34,580, Thermo Scientific) and quantified with an ImageQuant LAS 4000 mini platform (GE Healthcare). As loading control we used anti-α tubulin (1:3333; T9026-2ml, Sigma-Aldrich) and a secondary goat-anti-mouse antibody (1:10 000; sc-2005, Santa Cruz Biotechnology). Membranes were stripped prior to loading controls using stripping buffer (for 50 ml: 0.38 g glycine, 0.74 g sodium chloride, 350 µl of 14.3 M β-mercaptoethanol).

### RNA isolation, RNA-Seq and bioinfomatic analysis

RNA for Next Generation sequencing (NGS) was isolated using Maxwell® RSC Simply RNA Tissue kit (AS1340, Promega) according to manufacturer’s instructions. NGS libraries were prepared from 10 ng total RNA with Illumina Stranded total RNA Prep kit (#20040529, Illumina). NGS was performed on a NextSeq 500 or 550Dx instrument using indexed, 75 cycles single-end read protocol and a NextSeq 500/550 High Output Kit v2.5 (#20024906, Illumina). For analysis of NGS data we used freely available, customizable tools and a workstation. Image analysis and base calling resulted in .bcl files, which were converted into .fastq files by the bcl2fastq2 tool v2.17.1.14. and were mapped to human genome assembly GRCh38.87 v102 using HiSat2 Mapper, allowing one mismatch [38]. All unique hits were processed with featureCounts v2.0.1 [41]. Reads were counted locus-based, i.e. for unions of exons per gene. Batch effects caused by different sequencing runs, were removed with Combat Seq of SVA package using a negative binomial regression model that retains the integer nature of count data [77]. Principal component (PCA) and differential expression analyses were done within R v4.1.2 (R Core Team 2021) using DeSeq2 [46]. Volcano plots and heatmaps were generated with Enhanced Volcano R package [11], and Complex Heatmap R package [27, 28]. Functional annotation analyses were done based on differentially expressed genes (logFC ± 1, padj ≤ 0.05) with FUMA v1.4.1 [73] and Reactome release 82/Pathway browser 3.7 [25, 35]. Gene set enrichment analyses and single sample gene set enrichment analyses were performed with DeSeq2 normalized expression values using the GSEA module v20.3.5 [50, 66] or the ssGSEA v10.0.11 module [5, 66] on Gene Pattern [59], respectively. Gene sets for GBM expression subtypes were used from [13, 72], gene sets defining four main cellular GBM states were used from [52] and hallmark gene sets and oncogenic signature gene sets were used from [42]. Genes specifying mesenchymal-like (MES-like) tumor/immune cell interactions were used from [30]. For single cell deconvolution we used dREG [20] and single cell expression values (transcripts per million/TPMs) of an open-access IDH-wildtype GBM reference (GEO access number: GSE131928). Fastq.files and raw count tables of our analyzed samples were deposited in GEO (GEO access number: GSE230453).

### Statistics

We performed functional NGS analysis using R Statistical Software (v4.1.2; R Core Team 2021) and *in silico* analyses with R Statistical Software v3.5.3 and v4.0.2 (R Core Team 2021). We used SPSS v25/v28 and GraphPad Prism version v9 for descriptive statistics. For calculating significant differences non-parametric tests were used (Mann-Whitney U, post hoc Games-Howell) besides ordinary ANOVA with Tukey’s multiple comparison test. For correlation analyses, we used spearman rho methods. Significances are displayed as follows in figures: p > 0.05 = n.s., p < 0.05 = *, p < 0.01 = **, p < 0.001 = ***.

## Results

### Elevated *TSPO* mRNA expression in malignant gliomas is inversely correlated with promotor methylation

**Fig. 1:**
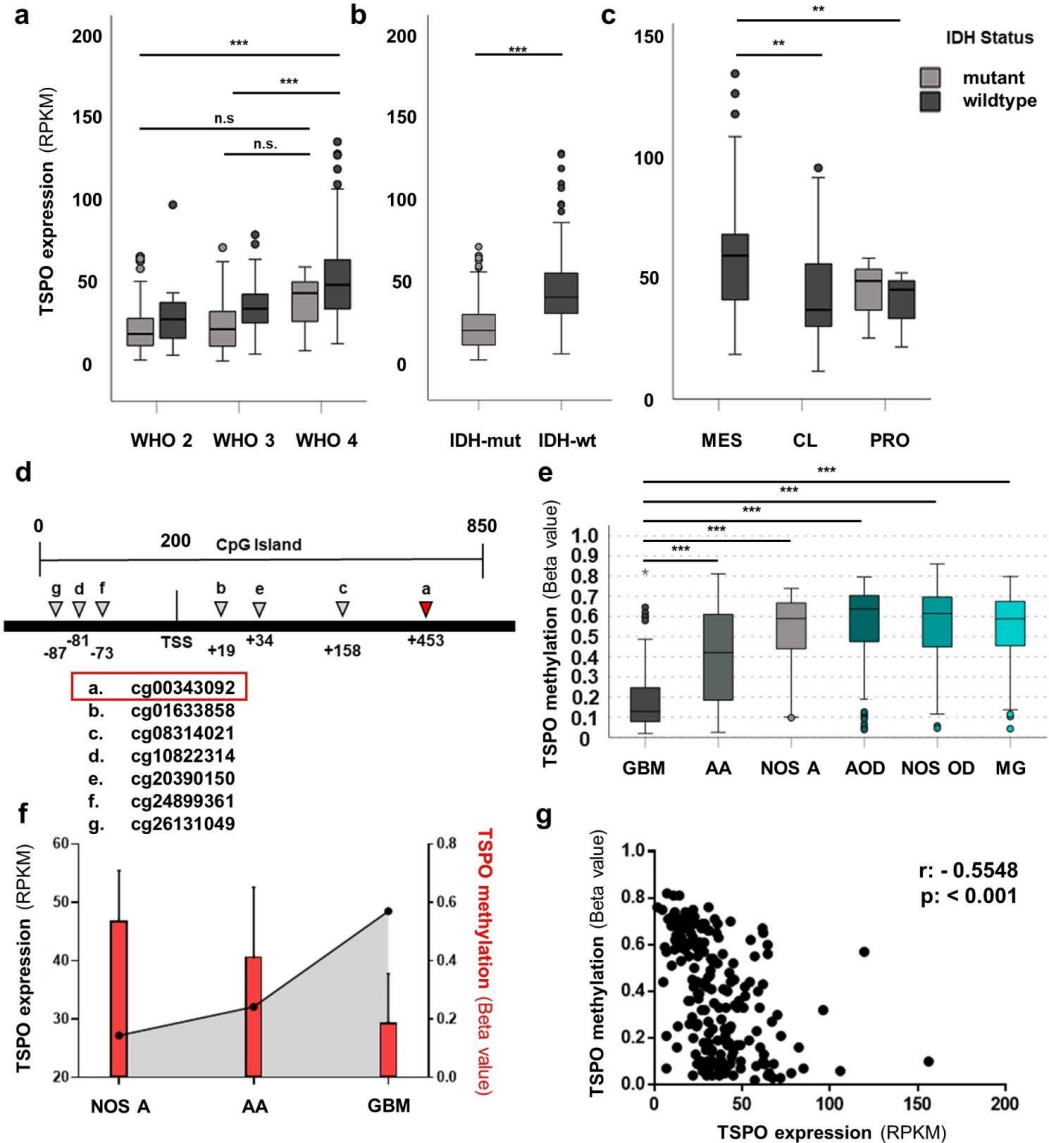
*TSPO* mRNA expression in malignant gliomas is inversely correlated with promotor methylation. *TSPO* mRNA expression (RPKM) was analyzed in a TCGA-GBM and TCGA-LGG *in silico* data set **(a-c)**. *TSPO* expression was analyzed based on WHO grades (WHO 2: 18 IDH-wt, 188 IDH-mut; WHO 3: 66 IDH-wt, 161 IDH-mut; WHO 4: 127 IDH-wt, 6 IDH-mut). Post-hoc Games Howell test revealed a significant increase of *TSPO* expression in WHO grade 4 gliomas (***p < 0.001) in comparison to WHO grade 2 and 3 **(a)**. Analysis based on *IDH* mutation status (IDH-mut: 355, IDH-wt: 211) showed a significantly higher *TSPO* expression in IDH-wt gliomas compared to IDH-mut gliomas (Mann Whitney U, ***p ≤ 0.001) **(b)**. Comparing expression subtypes of WHO grade 4 GBMs (MES: 56 IDH-wt; CL: 43 IDH-wt; PRO: 12 IDH-wt, 4 IDH-mut) *TSPO* expression was significantly higher in mesenchymal GBMs than in classical and proneural GBMs (Post Hoc Games Howell, **p < 0.01) **(c)**. A schematic diagram of *TSPO* promotor CpG island location on Ensembl GRCh38 reference genome covered by seven probes (a-g) on the Infinium® HumanMethylation450 Bead Chip and their distance to the TSS of the TSPO main transcript ENST00000337554 **(d)**. Statistical methylation analysis (Post Hoc Games Howell) of Beta values was performed with the TCGA-GBM and TCGA-LGG cohort for probe cg00343092 (marked in red) revealing significantly lower methylation levels in GBM (n = 130) compared to all other groups (AA: n = 129, NOS A: n = 62, AOD; n = 79, NOS OD: n = 118 and MG: n = 130) **(e)**. Mean TSPO CpG island methylation on probe cg00343092 (Beta value) and mean *TSPO* mRNA expression (RPKM) show an inverse correlation **(f)**. Spearman rho correlation of each matched value (n = 209) shows a significant inverse correlation (r = − 0.5548, ***p < 0.001) between methylation (probe cg00343092) and *TSPO* mRNA expression **(g)**. Significances are displayed as follows: p > 0.05 = n.s., p < 0.05 = *, p < 0.01 = **, p < 0.001 = ***. CL: classical, CpG: 5’-C-phosphate-G-3’, IDH: isocitrate dehydrogenase, IDH-wt: IDH-wildtype, IDH-mut: IDH-mutant, GBM: glioblastoma, LGG: low-grade glioma, MES: mesenchymal, PRO: proneural, RPKM: reads per kilobase per million, TCGA: The Cancer Genome Atlas, TSS: transcription start site, AA: anaplastic astrocytoma WHO grade 3, NOS: not otherwise specified glioma WHO grade 2, AOD: anaplastic oligodendroglioma WHO grade 3, NOS OD: not otherwise specified oligodendroglioma WHO grade 2, MG: mixed glioma

Previous studies showed *TSPO* overexpression in gliomas compared to non-neoplastic brain tissue and suggested positive correlation of *TSPO* expression and glioma malignancy [14, 71]. However, the mechanism of upregulation of *TSPO* had not yet been deciphered. We therefore first retrieved *TSPO* expression data from open-access RNA-Seq databases to reproduce the above mentioned findings [16]. We employed *in silico* queries from the TCGA Research Network and compared the normalized expression values (RPKMs) of *TSPO* among IDH-mutant/-wildtype gliomas across reported WHO grades, and across glioblastoma (GBM) transcriptional subtypes. We found that *TSPO* expression values increased significantly with WHO grade in IDH-wildtype gliomas (p ≤ 0.001) and were higher in IDH-wildtype than in IDH-mutant gliomas p < 0.001) (Fig. 1a/b). Within GBM transcriptional subgroups, a significantly higher *TSPO* expression in the prognostically unfavorable mesenchymal subgroup [9, 34] compared to the classical (p = 0.004) or proneural (p = 0.002) groups was observed (Fig. 1c).

To analyze a potential epigenetic regulation mechanism of *TSPO* expression we analyzed *in silico* open-access data of Illumina K450 methylation arrays from different glioma types (TCGA-LGG and TCGA-GBM). Methylation beta values of 15 probes covering the *TSPO* promotor gene locus were extracted (Suppl. Figure 2a). Seven probes covering the CpG island showed lower methylation, so we focused on this area for further analyses (Fig. 1d). One out of seven probes (cg00343092) showed a significant lower methylation level in GBMs compared to anaplastic astrocytomas (intermediate beta values, p < 0.001) and to the other glioma types (high beta values, p < 0.001) (Fig. 1e). We then assessed a potential relation between the observed low methylation and high *TSPO* expression in GBMs by analyzing patients from which matched RNA-Seq and methylation data were available. Separated by entity, *TSPO* expression values (RPKMs) and beta values of cg00343092 were inversely correlated (Fig. 1f); spearman rho analysis confirmed statistical significance (r = -0.5548, p < 0.001) (Fig. 1g).

Regarding reported GBM methylation subtypes (e.g. RTK I, RTK II, and MES), no significant *TSPO* expression differences between subtypes could be found (Suppl. Figure 2b). In regard to methylation of probe cg00343092 across reported GBM methylation subtypes, the MES group (n = 14) showed a significantly lower methylation (p = 0.044) than the RTK II group (n = 19) (Suppl. Figure 2c). Otherwise, no correlation of *TSPO* expression (RPKMs) or *TSPO* methylation (beta values) could be found to any of the GBM methylation subtypes (Suppl. Figure 2d).

To explore alternative regulatory mechanisms of *TSPO* expression we further analyzed large DNA-based glioma data sets (in total 531 samples) in cBioportal [17, 23] but neither detected any relevant *TSPO* coding mutations nor *TSPO* gene amplifications (data not shown).

### *TSPO* promotor hypermethylation depends on *IDH* mutation and is inversely correlated to *TSPO* expression

**Fig. 2:**
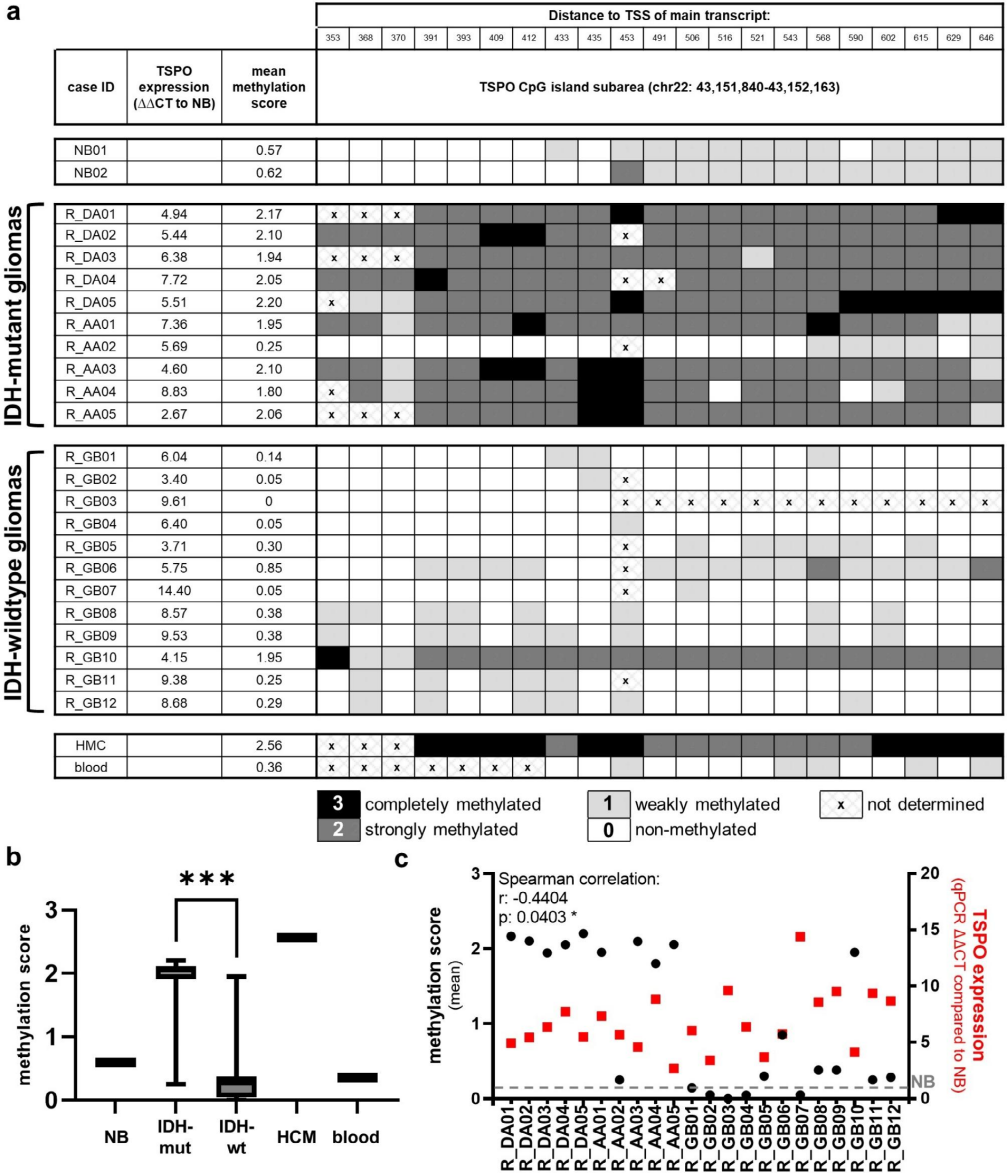
*TSPO* promotor hypermethylation as uncovered by direct bisulfite sequencing is correlated to *IDH*mutation. Overview of methylation scores from direct bisulfite sequencing and qPCR expression values for *TSPO* in IDH-mut (R_DA01-R_AA05) and IDH-wt gliomas (R_GB01-R_GB12) in *TSPO* CpG island subarea chr22: 43,151,840 − 43,152,163 **(a)**. Distribution of mean methylation scores reveals significant differences between IDH-mut and IDH-wt gliomas (Mann Whitney U, ***p < 0.001). Non-neoplastic brain shows weak/no methylation comparable to IDH-wt gliomas **(b)**. Spearman correlation of methylation scores and qPCR expression values reveals a weak but significant inverse correlation (r = − 0.4404, *p = 0.0403) **(c)**. Significances are displayed as follows: p > 0.05 = n.s., p < 0.05 = *, p < 0.01 = **, p < 0.001 = ***. Blood: unmethylated control, CpG: 5’-C-phosphate-G-3’, IDH: isocitrate dehydrogenase, HMC: hypermethylated control, IDH-wt: IDH-wildtype, IDH-mut: IDH-mutant, NB: non-neoplastic brain, TSS: transcription start site, qPCR: real-time quantitative polymerase chain reaction, R_DA: diffuse astrocytoma, R_AA: anaplastic astrocytoma, R_GB: glioblastoma

To better understand the mechanism of TSPO regulation, we extended methylation analyses beyond the 7 CpGs covered by the Illumina K450 methylation array. We performed direct bisulfite sequencing of nearly the complete *TSPO* promotor CpG island region (72 of 80 single CpGs covered) within 22 human glioma samples (10 IDH-mutant and 12 IDH-wildtype). We further analyzed *TSPO* expression by qPCR. Using JASPAR for the whole *TSPO* promotor CpG island region, we predicted binding sites for transcription factors (ETS1/2, SP1/2, and STAT3) known to be involved in TSPO regulation [6]. We observed various possible binding sites for ETS1/2 and SP1/2 along the CpG island, and fewer sites for STAT3. Bisulfite PCR revealed areas with strong methylation in IDH-mutant gliomas compared to a weak or absent methylation in IDH-wildtype gliomas (Suppl. Table 1). Differences were especially pronounced in a *TSPO* CpG island subarea (chr22: 43,151,840 − 43,152,163) around the probe cg00343092 detected in the *in silico* dataset (Fig. 2a). Within this subarea we observed accumulated binding sites for ETS1/2. Mean methylation scores in the identified *TSPO* CpG island subarea were high in IDH-mutant and low or absent in IDH-wildtype gliomas (p < 0.001) (Fig. 2b). Spearman rho correlation of mean methylation scores and *TSPO* expression values (ΔΔCT) in the same tumors revealed a weak but significant inverse correlation (r = − 0.4404, p = 0.0403) (Fig. 2c).

Of note, non-neoplastic brain tissue also showed a low/absent methylation within the identified *TSPO* CpG island subarea comparable to that of IDH-wildtype tumors (GBMs). This observation leads to an enhanced understanding of the observed methylation changes: We did not recognize hypomethylation in GBMs but a *de-novo* methylation in IDH-mutant tumors. Thus, overexpression of *TSPO* in gliomas is not induced by a loss of *TSPO* promotor methylation, but *TSPO* promotor subarea hypermethylation might serve as a mechanism to reduce *TSPO* expression levels in IDH-mutant compared to IDH-wildtype gliomas.

### TSPO antibody validation showed a specific staining pattern with no unspecific binding

We thoroughly tested the monoclonal TSPO antibody with western blot experiments on transient TSPO-knockdown glioma cells, on glioma cells with an antibody epitope blocking approach and on protein lysates from 4 different cryo-conserved GBM samples. Additionally, IHC was performed on TSPO-knockout microglia and an anaplastic astrocytoma with and without antibody epitope blocking. Western blots of glioma cells revealed a decrease of the antibody-binding signal in the transient TSPO-knockdown and a single band at the expected TSPO size (18 kDa). Another tested polyclonal TSPO antibody revealed unspecific binding patterns and was not used further (Suppl. Figure 3a). Epitope blocking resulted in a complete loss of the 18 kDa TSPO band in glioma cells (Suppl. Figure 3b) and a single band at the expected TSPO size was observed when glioblastoma protein lysates were analyzed (Suppl. Figure 3c). IHC staining showed TSPO expression in *TSPO*-wildtype microglia and no staining in TSPO-knockout microglial cells (Suppl. Figure 3d). Blocking of the epitope binding site resulted in a complete loss of IHC staining in an astrocytoma sample (Suppl. Figure 3e). In summary, all these experiments clearly demonstrate a specific staining pattern for the applied TSPO antibody with no indications for unspecific binding.

### TSPO-IHC correlates with TSPO-PET signal and is highest in the tumor core of GBMs

**Fig. 3:**
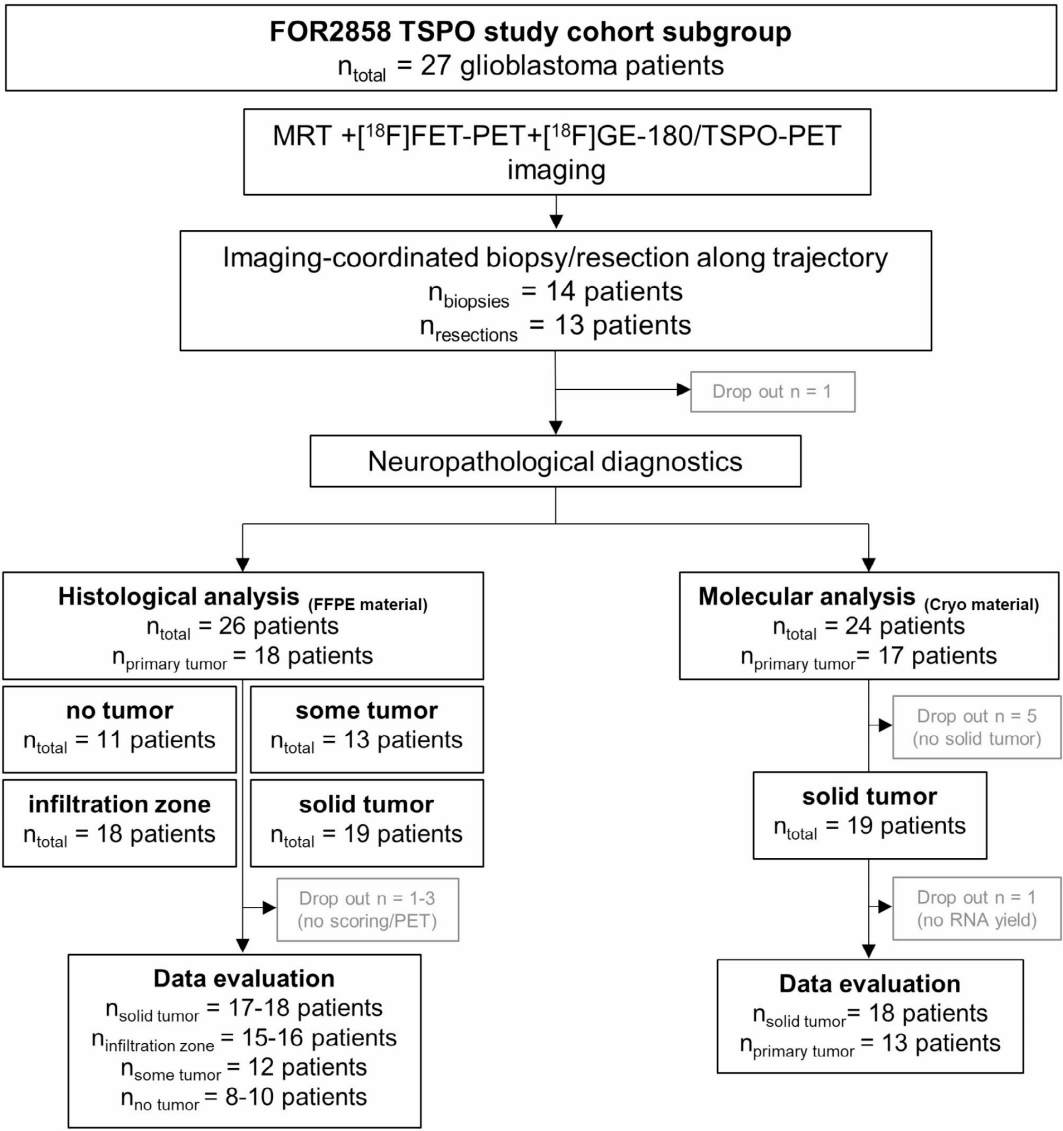
CONSORT flow diagram of the FOR2858 TSPO study (tissue-based aspects). The FOR2858 TSPO study cohort included in total 27 patients with GBM. All patients had received contrast-enhanced MRI, TSPO-PET and amino acid PET. Areas of interest were defined in an interdisciplinary exchange between the attending neurosurgeon and nuclear medicine specialist. Imaging-coordinated biopsy/resection along trajectory resulted in 14 patients with biopsy material and 13 with resection material for further analyses. Formalin-fixed and paraffin-embedded (FFPE) material from 26 patients with IDH-wildtype GBM (18 primary and 8 recurrent tumors) was used for further histological/molecular analyses. Tumor cell content was assessed by using H&E stains of each specimen with subdivision into the following categories: “no tumor”, “some tumor”, “infiltration zone”, “solid tumor” (see text for further explanations). In our stereotactical approach we collected multiple tissue samples from each individual patient that could match to different tumor cell content categories (for more detailed information compare Suppl. Table 2). After some dropouts, where no reliable immunohistochemical staining or no TSPO-PET value extraction was possible, 17–18 patients with solid tumor samples, 15–16 patients with infiltration zone samples, 12 patients with “some tumor” samples and 8–10 patients with no tumor were available for data evaluation (depending on the respective comparisons). For molecular analyses, fresh-frozen cryo material from 24 patients with IDH-wildtype GBM was collected in addition to the FFPE material. After exclusion of specimens with no solid tumor (5 patients) and specimens without sufficient RNA yield (1 patient) a subset of 18 patients with IDH-wildtype GBM (13 primary and 5 recurrent tumors) was available for molecular data evaluation.

For histopathological evaluation of TSPO as a PET imaging marker, we utilized IDH-wildtype glioblastoma (GBM) samples (n = 26) of our multidisciplinary prospective cohort [58]. All study patients underwent TSPO-PET imaging and targeted biopsy or resection for correlation of imaging with histological parameters (Fig. 3). Biopsy specimens were available from 14 and resections from 13 patients. One resection sample could not be further histologically analyzed due to insufficient tissue quality. For all collected tissue samples from 26 patients (18 primary tumors) TSPO-PET imaging values were extracted, histological classification of tumor cell content (solid tumor, infiltration zone, some tumor and no tumor) was performed and TSPO protein expression was analyzed immunohistochemically. In our stereotactical approach we collected multiple tissue samples from each individual patient that could match to different tumor cell content categories (for detailed information compare Suppl. Table 2). Solid tumor was collected from 19 patients (45.0% of all samples), the tumor infiltration zone was sampled in 18 patients (28.5%), areas with even lower tumor cell content (cortex and satellitoses or only sporadic infiltrating tumor cells, “some tumor”) were present in 13 patients (19.9%) and from 11 patients (6.6%) we retrieved material not containing histologically visible tumor (“no tumor”).

**Fig. 4:**
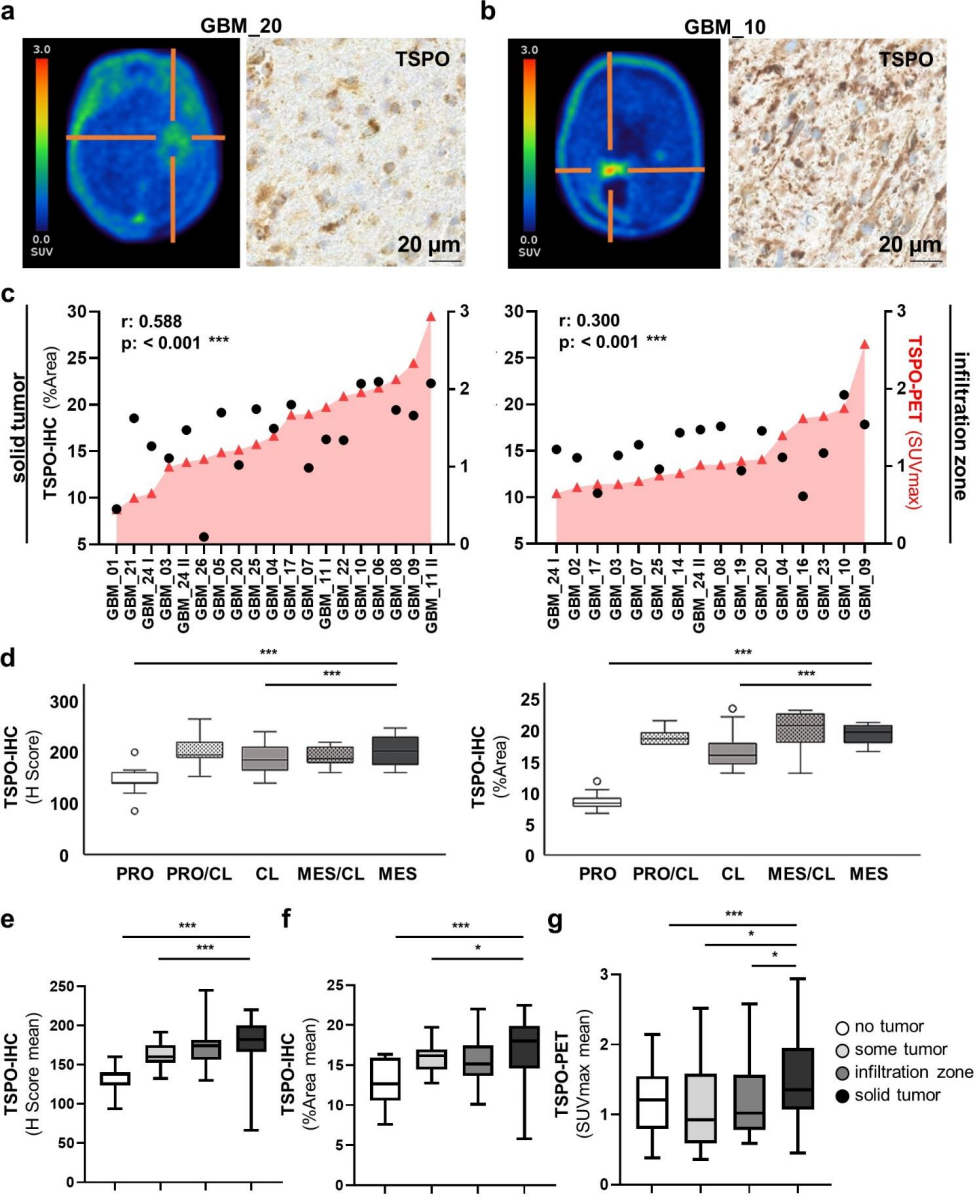
TSPO-IHC correlates to TSPO-PET signal and is highest in the solid tumor core of IDH-wildtype GBMs. TSPO-PET image and TSPO-IHC (400x) of a TSPO-PET low glioblastoma case (GBM_20) **(a)** and a TSPO-PET high glioblastoma case (GBM_10) **(b)**, further imaging information and full histology can be found in Suppl. Figure 4. Spearman correlation of TSPO-IHC labelling (%Area) with TSPO-PET enrichment (SUVmax) of IDH-wt GBMs showed an association with TSPO-PET enrichment in both solid tumor areas (r = 0.588, ***p < 0.001) and infiltration zones (r = 0.300, ***p < 0.001) **(c)**. TSPO H Score and TSPO-IHC %Area **(d)** in solid tumor areas weighted for sample amount per patient were compared between GBM expression subtypes. A significantly higher TSPO expression was observed in patients with mesenchymal (MES) transcriptome patterns compared to patients with proneural (PRO) or classical (CL) patterns (Post hoc Games Howell, ***p < 0.001). TSPO H Score **(e)**, TSPO-IHC %Area **(f)** and TSPO-PET enrichment values **(g)** were analyzed across different tumor cell content areas and solid tumor areas with the highest tumor cell content in all comparisons exhibited the highest TSPO signal (expression/enrichment), (Tukey multiple comparison test, ***p < 0.001, *p < 0.05). Significances are displayed as follows: p > 0.05 = n.s., p < 0.05 = *, p < 0.01 = **, p < 0.001 = ***. GBM: glioblastoma, IDH: isocitrate dehydrogenase, IDH-wt: IDH-wildtype, IHC: immunohistochemistry, PET: positron-emission tomography, SUVmax: maximum standardized uptake value

Inter- as well as intra-tumoral heterogeneity was observed for TSPO-PET imaging and TSPO-IHC. Figure 4a/b provides visualization of one patient with low TSPO-PET signal (GBM_20) and one patient with high TSPO-PET signal (GBM_10) together with the corresponding TSPO-IHC. The case with low TSPO-PET tracer uptake showed a weak TSPO staining in IHC, whereas the case with high TSPO tracer uptake showed a strong TSPO staining. Imaging information (TSPO-PET, FET-PET and MRI) and full histology (H&E and TSPO-IHC) for both cases is supplied in Suppl. Figure 4. To further analyze TSPO-PET imaging correlation to TSPO-IHC, we compared TSPO-PET imaging SUVmax values with their corresponding TSPO-IHC %Areas. We used all specimens with solid tumor (73 biopsy/resection specimens from 17 patients) and infiltration zone (50 biopsy/resection specimens from 15 patients). TSPO-IHC and TSPO-PET (separated by patient) showed a clear positive correlation between both parameters (Fig. 4c). Statistical significance was confirmed by a sample-to-sample spearman rho analysis within the respective tumor content cell group weighted per patients’ sample numbers (solid tumor: r = 0.588, p < 0.001; infiltration zone: r = 0.300, p < 0.001). Of note, when comparing TSPO protein expression across GBM expression subtypes (Fig. 4d), we found a significantly higher TSPO expression in the solid tumor areas of patients with mesenchymal transcriptome patterns compared to patients with proneural or classical patterns (p < 0.001, both with an intensity-based H score and %Area score).

We also compared TSPO signal intensity across the different tumor cell content groups. TSPO-IHC staining intensity increased with increasing tumor cell content. Analyzing the mean distribution of TSPO-IHC semi-quantified both with an intensity-based H score and %Area score in all 26 patients we found a significantly higher TSPO expression in the solid tumor areas compared to areas containing some tumor cells or no tumor at all (p < 0.05, p < 0.001) (Fig. 4e/f). Analyzing TSPO-PET (SUVmax) in the same way, we also found a significantly higher TSPO signal in the solid tumor area compared to all other areas with lower tumor cell content (p < 0.001, p < 0.05) (Fig. 4g). Thus, the highest overall TSPO signals were observed in the solid tumor core regions with a high glial tumor cell content.

### TSPO is expressed by diverse cell populations and CD68-positive macrophages/microglia drive TSPO signal in the infiltration zone

**Fig. 5:**
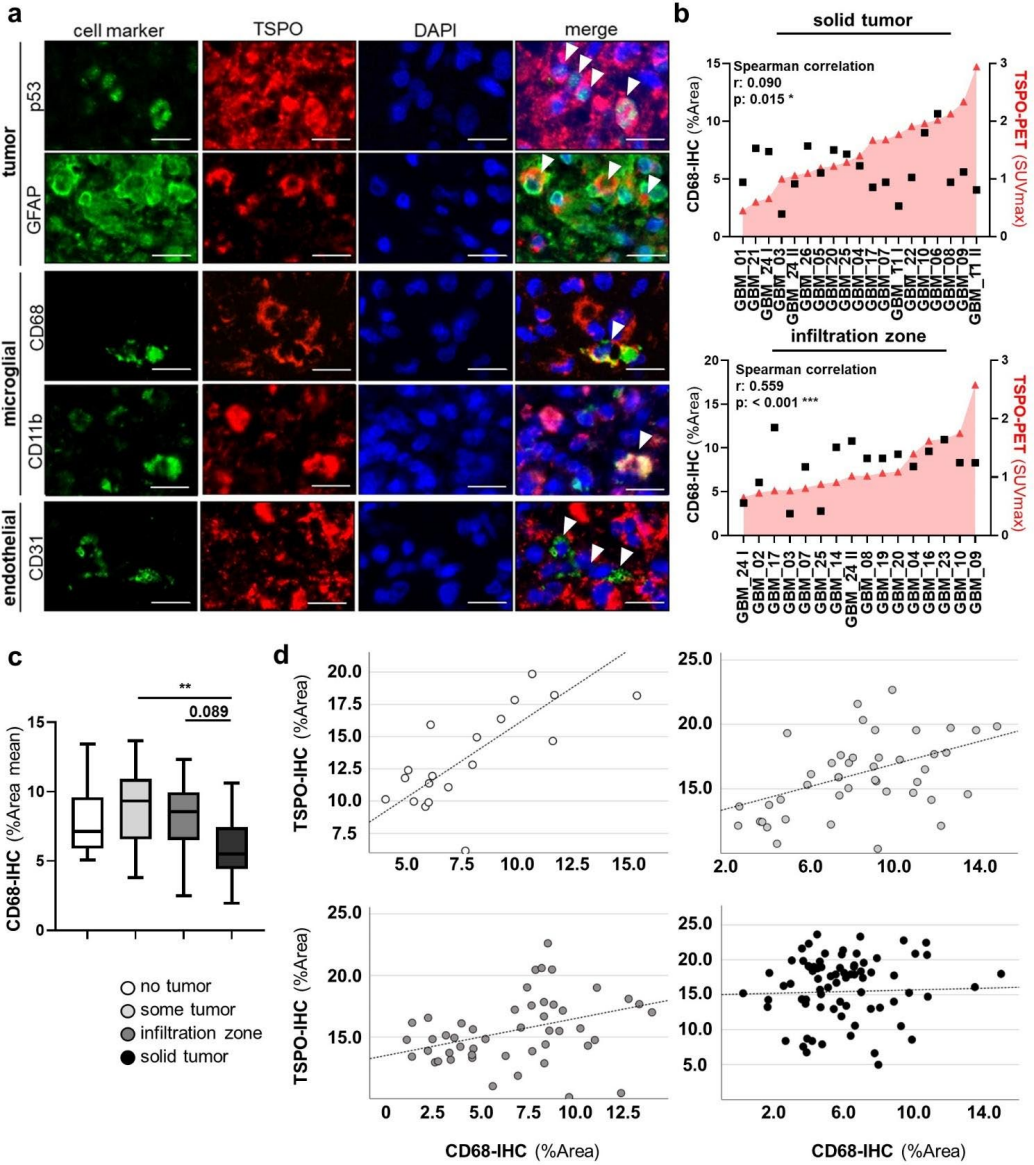
TSPO is expressed in glial tumors by diverse cell populations and CD68-positive macrophages/microglia drive TSPO expression in the tumor infiltration zone. Multiplex OPAL immune fluorescence co-staining (scale bar = 20 μm) of TSPO together with p53 (tumor cell marker), GFAP (astrocytic differentiation marker), CD68/CD11b (microglia/macrophage marker), and CD31 (endothelial cell marker) in a TSPO-PET enriched IDH-wt glioblastoma patient (GBM_11 II) showed that TSPO is expressed in glial tumors by diverse cell populations **(a)**. Spearman correlation of CD68-IHC labelling (%Area) with TSPO-PET enrichment (SUVmax) in solid tumor areas showed almost no association (r = 0.090, *p = 0.015), whereas in infiltration zones a clear association (r = 0.559, ***p < 0.001) was observed **(b)**. Comparison of the mean distribution patterns of CD68-IHC %Area values across different tumor cell content groups showed lowest signals in solid tumors regions, (Tukey multiple comparison test, **p < 0.01) in comparison to tumor-adjacent regions **(c)**. Spearman correlation of TSPO-IHC with CD68-IHC (%Areas) for no tumor (white), some tumor (light grey), infiltration zone (dark grey), and solid tumor (black) revealed strongest TSPO/CD68-IHC association in specimens without histological tumor characteristics (r = 0.686, ***p < 0.001), whereas with increasing content of glial tumor cells association decreases (some tumor: r = 0.414, ***p < 0.001, infiltration zone: r = 0.403, ***p < 0.001, solid tumor: r = 0.027, p = 0.472) **(d)**. Significances are displayed as follows: p > 0.05 = n.s., p < 0.05 = *, p < 0.01 = **, p < 0.001 = ***. CD11b: cluster of differentiation 11b/ integrin alpha M, CD31: cluster of differentiation 31/ platelet and endothelial cell adhesion molecule 1, CD68: cluster of differentiation 68/ macrosialin, GBM: glioblastoma, GFAP: glial fibrillary acidic protein, IDH: isocitrate dehydrogenase, IDH-wt: IDH-wildtype, IHC: immunohistochemistry, p53: tumor protein p53, PET: positron-emission tomography, SUVmax: maximum standardized uptake value

To further analyze the cellular source of TSPO, immunofluorescence co-staining for TSPO with cell type differentiation and tumor-markers was performed in a TSPO-enriched patient with IDH-wildtype GBM (GBM_11 II). As described in the literature, microglia/macrophages, endothelial cells and tumor cells can be a cellular source for TSPO [33, 53, 60, 79]. We used p53/GFAP for staining astrocytic tumor cells, CD11b/CD68 for microglia and macrophages [36], and CD31 for endothelial cells [43] (Fig. 5a). Double staining revealed that on a single cell level in tumor core regions TSPO was expressed by all these cell populations (i.e. p53/GFAP-positive astrocytic tumor cells, CD68/CD11b-positive microglia/macrophages and CD31-positive endothelial cells).

As microglia/macrophages are often described cellular sources of TSPO [22, 79], we stained all 26 patients with IDH-wildtype GBM with a well-established CD68 [PG-M1] antibody. When correlating CD68-IHC with TSPO-PET in the same way as we did for TSPO-IHC, we observed clear differences (Fig. 5b). While there was almost no association between TSPO-PET imaging and CD68-IHC (Spearman correlation: r = 0.090, p = 0.015) in the solid tumor areas, there was a weak association between TSPO-PET and CD68-IHC in the infiltration zones (Spearman correlation: r = 0.559, p < 0.001). When analyzing CD68-IHC (%Area) across the tumor content groups, we also observed clear differences in CD68 expression with the lowest CD68 expression in the solid areas and higher CD68 expression in the tumor-adjacent areas (infiltration zone, p = 0.089, some tumor, p < 0.01) (Fig. 5c).

Given this finding, we further analyzed the dependency of TSPO/CD68 expression by performing direct spearman correlation analyses between TSPO-IHC (%Area) and CD68-IHC (%Area) in the different tumor cell content groups (Fig. 5d). We observed a decrease/loss of association of TSPO expression and CD68 expression with increasing tumor cell content (no tumor: r = 0.686, p < 0.001; some tumor: r = 0.414, p < 0.001; infiltration zone: r = 0.403, p < 0.001; solid tumor: r = 0.027, p = 0.472). Thus, we conclude that CD68-positive microglia/ macrophages are a relevant source of TSPO expression/enrichment, predominantly in tumor-adjacent zones and less in solid tumor core areas.

### *TSPO* overexpression marks oncogenic signaling, extracellular matrix organization and immune system interaction patterns

To better understand the molecular changes marked by *TSPO* expression in GBM, we analyzed fresh-frozen specimens from solid tumor areas of our TSPO-PET imaging study patients (n = 18, tumor content > 80%) by RNA-Seq (Fig. 3, Suppl. Table 3). We assured that *TSPO* mRNA expression (RNA-Seq, Cryo tissue, DeSeq normalized counts) significantly correlated with TSPO protein expression (IHC, FFPE tissue, TSPO %Area) (r = 0.504, p = 0.04) (Suppl. Figure 5a).

**Fig. 6:**
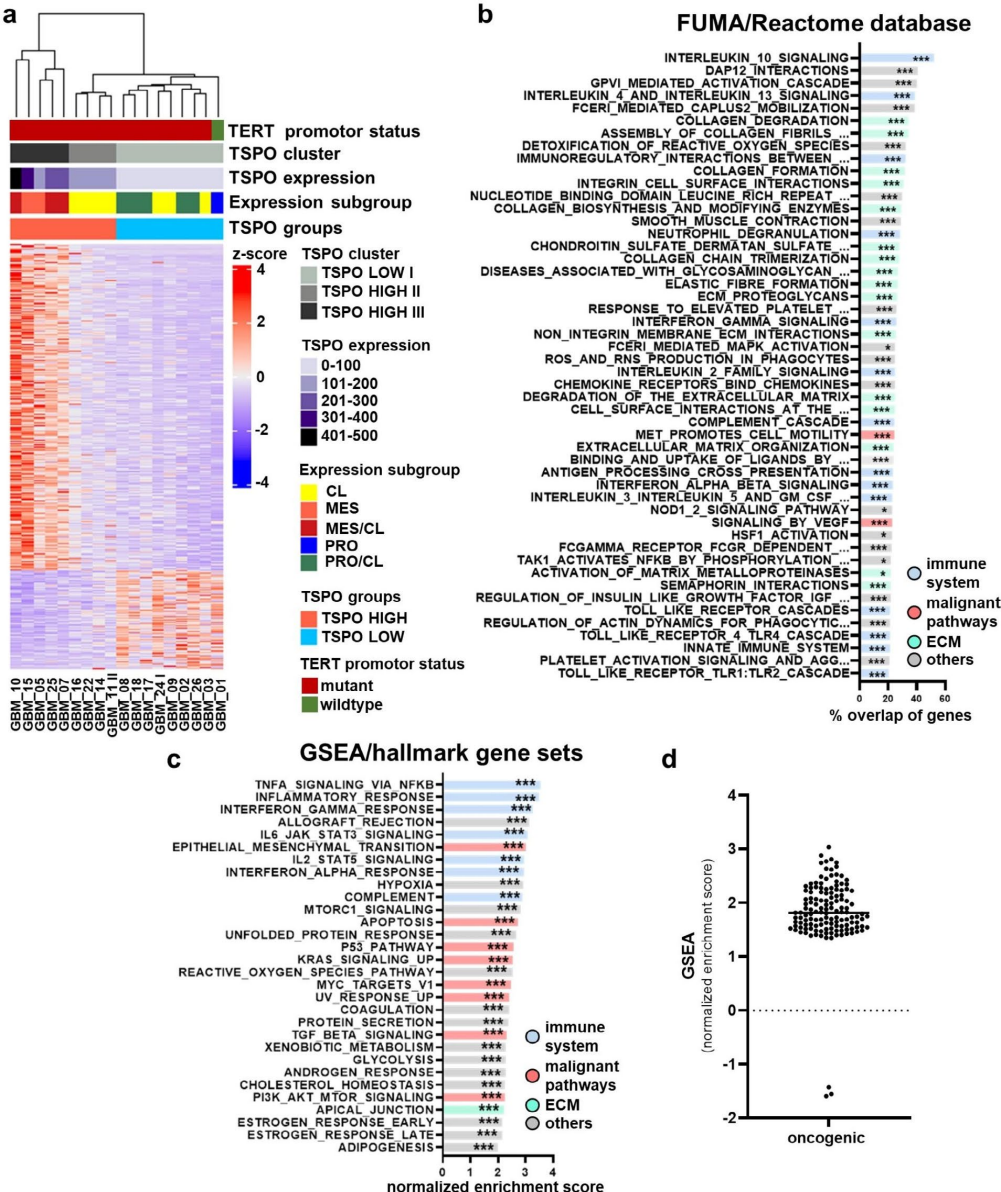
*TSPO* overexpression marks oncogenic signaling, extracellular matrix organization and immune system interaction patterns. Heatmap with annotation and unsupervised clustering of differentially expressed genes of 9 *TSPO*-low vs. 9 *TSPO*-high GBM cases analyzed with RNA-Seq (*TSPO* mRNA expression median split) **(a)**. Follow-up analyses in Reactome/ FUMA with the differentially expressed genes predominantly showed three functional clusters among the top 50 overrepresented pathways (Reactome: FDR ≤ 0.25, FUMA: padj ≤ 0.05): ECM organization, immune system interaction, and malignant/oncogenic pathways **(b)**. GSEA using Hallmark gene sets revealed 30 significant gene sets (FDR ≤ 0.05, NES > 2.0) from these three functional clusters **(c)** and an enrichment of oncogenic signature transcripts in *TSPO*-high cases (FDR ≤ 0.05, FWR ≤ 0.05) **(d)**. Significances are displayed as follows: p > 0.05 = n.s., p < 0.05 = *, p < 0.01 = **, p < 0.001 = ***. CL: classical, ECM: extracellular matrix, FDR: false discovery rate, FUMA: Functional Mapping and Annotation, FWR: familywise-error rate, GBM: glioblastoma, GSEA: gene set enrichment analysis, IDH: isocitrate dehydrogenase, IDH-wt: IDH-wildtype, MES: mesenchymal, NES: normalized enrichment score, PRO: proneural

For further analysis, we split the RNA-Seq data according to the median *TSPO* expression (median = 92.90) into a *TSPO*-low and a *TSPO-*high group. The distribution of normalized expression counts in the *TSPO-*low/high group is shown in Suppl. Figure 5b. Differential expression analysis between both groups revealed that 1581 genes in total were differentially expressed (logFC ± 1, padj < 0.05), with 1213 genes upregulated and 368 genes downregulated (Fig. 6a). In subsequent functional annotation analyses (Reactome and FUMA), mostly the upregulated genes led to significant overrepresentation hits. The top 50 overrepresented pathways in the FUMA/Reactome database using all differentially expressed genes (Reactome: FDR ≤ 0.25, FUMA: padj ≤ 0.05) were mainly related to three functional clusters: extracellular matrix (ECM) reorganization/cell migration (16 pathways), immune system interaction (13 pathways) and oncogenic pathways (2 pathways) (Fig. 6b). Furthermore, the analysis of normalized expression values (DeSeq normalized expression values) with gene set enrichment analysis (GSEA) revealed 30 significantly enriched hallmark gene sets (FDR ≤ 0.05, NES > 2.0) mainly from those functional clusters (Fig. 6c). Eight gene sets were involved in immune system interaction, 6 gene sets were indicative of higher tumor malignancy and 1 gene set was related to extracellular matrix organization. Additionally, we performed GSEA with gene sets for oncogenic signatures (Fig. 6d) and found 136 significant enriched pathways (FDR < 0.25). The vast majority of the gene sets (133) were enriched in the *TSPO-*high group, clearly indicating a more malignant transcriptional phenotype in the *TSPO-*high group. Taken together, our results suggest that tumor regions with high *TSPO* expression are highly malignant and exhibit a pronounced tumor-immune system interaction and extracellular matrix organization.

### High *TSPO* expression marks mesenchymal glioblastoma cell subpopulations characterized by elevated numbers of tumor-associated macrophages

**Fig. 7:**
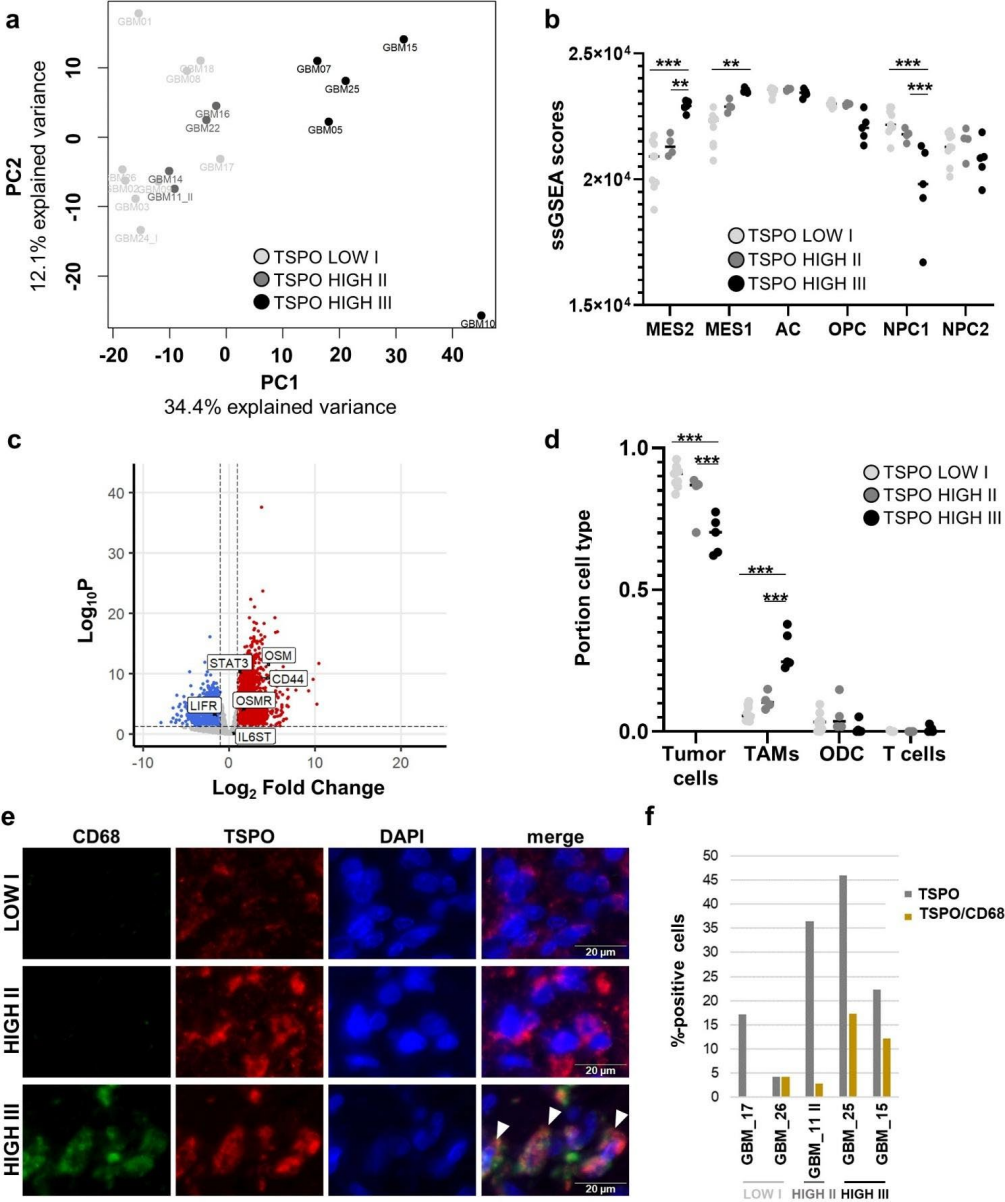
High *TSPO* expression marks mesenchymal glioblastoma cell subpopulations characterized by elevated numbers of tumor-associated macrophages. Principal component analysis of RNA-Seq data showed a separation of a cluster (TSPO HIGH III) consisting of all mesenchymal GBMs from the non-mesenchymal GBMs (TSPO LOW I, TSPO HIGH II) **(a)**. ssGSEA with gene sets defining cellular states in GBM [52] revealed an enrichment of the MES1/2-like cellular states in the HIGH III cluster of the *TSPO*-high group **(b)**. Gene expression of MES-like TAM tumor cell interaction genes [30] is upregulated in the TSPO HIGH III compared to the TSPO LOW I cluster **(c)**. Deconvolution of bulk sequencing data shows a significant larger TAM cell proportion in TSPO HIGH III cluster in comparison to TSPO HIGH II and TSPO LOW I **(d)**. Representative images of OPAL multiplex immune fluorescence staining showed a higher TSPO/CD68-positive cell portion in the TSPO HIGH III (GBM_25) case in comparison to TSPO HIGH II (GBM_11 II) and TSPO LOW I (GBM_17) cases **(e)**, whole OPAL images and corresponding H&E staining can be found in Suppl. Figure 7. Quantifications (%positive cells) of TSPO-positive and TSPO/CD68-positive cell portion by counting of n = 2 TSPO LOW I, n = 1 TSPO HIGH II and n = 2 TSPO HIGH III cases verified those findings **(f)**. Significances are displayed as follows: p > 0.05 = n.s., p < 0.05 = *, p < 0.01 = **, p < 0.001 = ***. AC: astrocyte like, CD44: cluster of differentiation 44, CD68: cluster of differentiation 68/ macrosialin, DAPI: 4′,6-diamidino-2-phenylindole, GBM: glioblastoma, IL6ST: GP130/ glycoprotein 130, LIFR: leukemia inhibitory factor receptor, MES1/3: mesenchymal-like, NES: normalized enrichment score, NPC1/2: neural progenitor like, ODC: oligodendrocytic cells, OPC: oligodendrocyte progenitor like, OSM: oncostatin M, OSMR: oncostatin M receptor, PC1/2: principal component dimension 1 and 2, STAT3: signal transducer and activator of transcription 3, ssGSEA: single sample gene set enrichment analysis, TAM: tumor-associated macrophages

When further characterizing the GBMs from our RNA-Seq analysis in terms of their transcriptional subtypes [13, 72], it was striking that all 5 GBM samples with the prognostically unfavorable mesenchymal subtype were in the *TSPO*-high group (Fig. 6a, Suppl. Figure 6a). Unsupervised clustering of differentially expressed genes revealed a separate cluster within the *TSPO*-high group consisting of these 5 GBMs with the mesenchymal subtype, and principal component analysis also showed a separated cluster with these 5 GBMs (Figs. 6a and 7a). Of note, these 5 GBM samples also showed the highest *TSPO* expression values (Fig. 6a, Suppl. Figure 6b). We, therefore, performed further analyses with the three clusters revealed by unsupervised clustering of differentially expressed genes (TSPO LOW I, TSPO HIGH II and TSPO HIGH III) (Figs. 6a and 7a).

It had been shown recently that single cell RNA-Seq analysis of a large GBM cohort resulted in four main cellular states: neural progenitor-like (NPC1/2-like), oligodendrocyte progenitor-like (OPC-like), astrocyte-like (AC-like) and mesenchymal-like (MES1/2-like) [52]. MES-like states were linked to *NF1* mutations and tissue microenvironment interaction and are more abundant in the mesenchymal expression subtype. We performed ssGSEA with all cellular state gene sets in our bulk RNA-Seq data and observed a significant enrichment of MES1/2-like genes in the TSPO HIGH III cluster (Fig. 7b). Hara and colleagues described that MES-like cellular states in GBM cells were promoted by macrophage-derived Oncostatin M (OSM) that interacts with its receptors (OSMR and LIFR) in complex with GP130 (also known as IL6ST) via STAT3 signaling pathways [30]. Indeed, in the TSPO HIGH III cluster we observed a significant overexpression of the MES-like TAM tumor interaction genes OSM, OSMR, STAT3 and of CD44 (Fig. 7c). Additional direct gene-to-gene expression correlations in our IDH-wildtype GBMs (TSPO study cohort, TPMs) revealed moderate to strong associations between expression of TSPO and MES-like cellular state markers (Suppl. Figure 6c). Thus, our RNA-Seq results clearly point towards a high immune system involvement in TSPO HIGH III cluster tumors.

We then performed deconvolution analysis with a single cell RNA-Seq dataset (scRNA-Seq) as basis for specific cell type genes: tumor cells, tumor-associated macrophages (TAMs), oligodendrocyte cells (ODC) and T cells [52]. Displaying *TSPO* expression for cell type clusters (malignant cells, macrophages, oligodendrocytes, and T cells) in this scRNA-Seq dataset demonstrated *TSPO* expression in all cell types (Suppl. Figure 6d). As shown in Fig. 7d, TSPO HIGH III cluster tumors showed a significant decrease in tumor cells and a significant increase in the TAM cell population patterns. Results could be confirmed when extending our previously performed double fluorescence stains for TSPO/CD68 in respect to the here defined *TSPO* clusters (TSPO LOW I, n = 2, TSPO HIGH II, n = 1 and TSPO HIGH III, n = 2). Representative images of all three clusters (GBM_17 = TSPO LOW I, GBM_11 II = TSPO HIGH II, GBM_25 = TSPO HIGH III) showed highest relative amount of CD68-positive macrophages/microglia in TSPO HIGH III group tumors (Fig. 7e). A quantitative analysis of cell populations in all stained samples confirmed a higher relative content of TSPO/CD68-positive macrophages/microglia in the TSPO HIGH III group tumors (Fig. 7f).

Taken together, our RNA-Seq results showed that the *TSPO-*high group consists of two clusters, whereof the cluster with the highest *TSPO* expression shows an enrichment of mesenchymal signatures and immune system genes and contains an altered cellular composition with higher relative amounts of TSPO/CD68-positive macrophages/microglia.

## Discussion

TSPO is frequently overexpressed in glioma [3, 14, 71, 79], and a possible connection between TSPO enrichment and high malignancy has been suggested. TSPO is intensely discussed as an imaging target for prognosis [14, 58], during therapy [22, 57, 62, 63], and in CNS pathologies with neuroinflammatory components [4, 18, 29, 78]. Nevertheless, systematic approaches to link TSPO imaging to its histopathological correlates that would add informational content on TSPO as a biomarker are largely missing. Furthermore, it is still unclear how TSPO expression is regulated in CNS neoplasia. To address these open questions we used large *in silico* datasets and precisely clinically annotated patient collectives from which we had matching TSPO-PET imaging data and tissue specimens for histological and molecular analyses.

Regarding TSPO regulation, initial *in silico* analyses and confirmation on an own cryo-conserved tissue collective established a role for *TSPO* promotor hypermethylation in the reduction of *TSPO* expression levels in the molecularly defined subgroup of IDH-mutant gliomas. Our finding of an epigenetic regulation of *TSPO* is in line with results reported in a Jurkat human T cell leukemia cell line where demethylation with 5-aza-2′-deoxycytidine caused a dose-dependent increase in *TSPO* mRNA [48]. It is also known that IDH-mutant gliomas commonly exhibit a genome-wide hypermethylation phenotype [10, 67] and the observed *TSPO* hypermethylation might be part of this. We found that the hypermethylated *TSPO* promotor subarea contains a number of potential transcription factor binding sites that might be blocked. One of these potentially affected transcription factors is ETS1/2, for which a transcriptional regulation of *TSPO* [6] and a binding inhibition through DNA methylation has already been described [32]. Own *in silico* analyses revealed substantial *ETS1* and *ETS2* expression in non-neoplastic brain and tumor tissue. *ETS1* was upregulated and *ETS2* downregulated in GBMs compared to non-neoplastic brain tissue. Correlation analysis of *TSPO* and *ETS* revealed a weak but significant association of *TSPO* and *ETS2* mRNA expression in GBMs *in silico* and in our own study cohort (Suppl. Figure 8). Of note, we found the *TSPO* promotor unmethylated in non-neoplastic brain tissue. Thus, the hypermethylation we report is a *de novo* methylation restricted to IDH-mutant gliomas. In glioblastomas*/*IDH-wildtype gliomas the *TSPO* promotor was equally unmethylated as in the non-neoplastic controls and we neither observed *TSPO* gene amplifications nor *TSPO* gain of function mutations. In this setting, transcription factor binding to an unmethylated *TSPO* promotor most likely explains the overexpression of *TSPO* in IDH-wildtype gliomas. In MA-10 Leydig cells it has been shown that PKC_ε_ regulates *TSPO* gene expression through MAPK (Raf-1-MEK1/2-ERK1/2)-mediated transcriptional activation [6] and we know that these pathways besides others are commonly dysregulated in glioblastoma [37, 55]. In summary, *TSPO* transcriptional regulation in gliomas might be the result of a complex interplay between changes in *TSPO* promotor methylation and their effects on transcription factor binding.

The current literature on TSPO as an imaging marker (for review see [40]) clearly strengthens the need for histopathological evaluation of TSPO imaging correlates. The high-affinity TSPO ligand [^18^ F]GE180 was first used by Albert and colleagues for TSPO-PET imaging of untreated and pretreated GBM and showed remarkably high tumor-to-background contrast and TSPO-PET signal even in areas without contrast-enhancement on MRI [2]. This study was then extended with more cases of IDH-wildtype/-mutant gliomas by Unterrainer and colleagues [68]. We now provide a concomitant histopathological evaluation of patients with GBM that underwent the [^18^ F]GE180 TSPO imaging protocol. First, we successfully showed that the TSPO-PET signal correlates with TSPO expression as detected by immunohistochemistry with a thoroughly validated antibody. Secondly, our results revealed that the TSPO signal originates from multiple cellular sources, including tumor cells, reactive astrocytes, microglia/ macrophages and endothelial cells. Assessment of the regional heterogeneity of TSPO revealed that solid tumor-cell-rich areas are the major contributors to the overall TSPO signal. Tumor-adjacent areas show a lower TSPO enrichment/expression. Of note, in these regions the TSPO signal is mainly driven by CD68-positive microglia/macrophages.

Currently, there is one other similar human study investigating TSPO imaging and histopathological features in combination [79]. However, this study used the [^18^ F]DPA-714 imaging tracer and a different spectrum of tumors (smaller total number: 9 vs. 26 patients in our study, focus on IDH-mutant gliomas, only one GBM vs. 26 in our study) so that the results may not be entirely comparable. Zinnhardt and colleagues found a strong relationship between [^18^ F]DPA-714 uptake and activation of glioma-associated myeloid cells. TSPO expression was mainly restricted to tumor-infiltrating HLA-DR^+^ myeloid-derived suppressor cells (MDSCs) and TAMs. These findings match our observations in the tumor-infiltration zone. However, we additionally describe a relevant degree of intratumoral heterogeneity with higher TSPO expression in the solid tumor core that is characterized by the highest tumor cell content. It appears indeed very likely that in our patient cohort there is a stronger contribution of tumor cells to the overall TSPO signal, as GBM/IDH-wildtype gliomas (as outlined above) have an unmethylated *TSPO* promotor and overall higher TSPO expression levels than the IDH-mutant gliomas studied in the Zinnhardt paper [79].

Our observation of divergent cell populations contributing to the TSPO signal is also reinforced by investigations on human or murine GBM implantation-based mouse models [14]. There, comparable to our results, TSPO expression was observed in tumor cells, microglia, tumor-associated macrophages and endothelial cells and the authors proposed a combination of TSPO-PET and FET-PET as a promising way to visualize tumor-associated myeloid cells.

Interestingly, another study on GBM mouse models described an increase of tracer uptake during temozolomide (TMZ) chemotherapy [22]. Our study contained a limited number of 8 recurrent GBM only, but we also observed higher TSPO uptake/expression in those tumors compared to primary, therapy-naïve GBMs. It is indeed very likely that the cellular composition might change under therapy (decrease in tumor cells, increase in reactive and myeloid cells). Therefore we plan to extend our human study to a longitudinal setting in order to increase the number of matched pairs of primary and recurrent tumors and to investigate changes in TSPO enrichment/expression in the course of the disease.

Important findings were revealed by the molecular characterization of our current study. By RNA-Seq we linked high *TSPO* expression to the three functional clusters “oncogenic signaling, immune system interaction and extracellular matrix organization”. We further found that high *TSPO* expression indicated the mesenchymal transcription subtype and MES-like cell populations, which are associated with a worse prognosis [9, 34] and a pronounced interaction of tumor cells with the immune system [30]. In line with our findings, various signaling pathways related to inflammation are upregulated in tumors with high *TSPO* expression [3]. TSPO can be either involved in an anti-tumor/pro-inflammatory setting with M1 type microglia/macrophages or a pro-tumor/anti-inflammatory setting with M2 type microglia/macrophages [76]. Our transcriptional analysis suggests a complex role of *TSPO* in tumor-associated inflammation. In tumors with high *TSPO* expression, on the one hand, we see an enrichment in IFN/TNF-signaling typical for M1 type microglia/macrophages. On the other hand, we observe an overrepresentation in IL-10/-4/-13 signaling genes reactivating the M2 type microglia/macrophages. Interestingly, M2 macrophages are significantly associated with the mesenchymal phenotype [72] where we see the highest *TSPO* expression.

By analyzing *in silico* samples from patients with GBM of the TCGA database, Cai et al. first described that *TSPO* is highly expressed in the prognostically unfavorable mesenchymal GBM transcriptional subtype [14]. We now could confirm this finding in our own patient cohort of the TSPO imaging study. In addition, we further linked high *TSPO* expression to recently described, MES-like cellular states [52] with their described induction patterns by macrophages [30] and high numbers of TAMs. Indeed, mesenchymal GBMs display the highest percentage of microglia, macrophage, and lymphocyte infiltration from all transcriptional GBM subtypes [47]. Interestingly, *TSPO* as a marker for MES-like cellular states might be of therapeutical interest. In view of their high interaction with immune cells, MES-like cells are potential emerging targets for immune checkpoint inhibition [74]. Moreover, *TSPO* could qualify as a predictive biomarker for TAM-targeting immunotherapy [19].

In conclusion, our study improves the understanding of TSPO as an imaging marker in gliomas. We describe a novel mechanism of TSPO silencing in IDH-mutant astrocytomas. The histological and molecular evaluation of 26 patients with GBM that underwent a defined TSPO imaging procedure provides novel insights into the intratumoral heterogeneity of the TSPO signal. While high signal intensities are observed in the tumor-cell-rich solid core regions, lower TSPO signals in the tumor rim are mostly driven by CD68-positive microglia/macrophages. Finally, we identify *TSPO* expression as an indicator for the presence of a prognostically unfavorable mesenchymal GBM cell subpopulation characterized by a higher amount of TAMs and pronounced immune system interactions.

### Electronic supplementary material

Below is the link to the electronic supplementary material.

**Supplementary Fig. 1 Representative image fusion example generated with Brainlab planning software for the FOR2858 TSPO-PET imaging study**. All patients received contrast-enhanced MRI, [^18^ F]GE180-PET and [^18^ F]FET-PET within a maximum of 18 and a median of 3 days before the operation. MRI included gadolinium enhanced T1- (1 mm slices) and T2‐weighted scans (2 mm slices). For [^18^ F]GE180-PET, approximately 180 MBq [^18^ F]GE180 were injected intravenously and summation scans 60–80 min post injection were used for image analysis. For [^18^ F]FET-PET, approximately 180 MBq [^18^ F]FET were injected and 40 min post injection summation images were analyzed as described previously [68]. Areas of interest were defined in an interdisciplinary exchange between the attending neurosurgeon and nuclear medicine specialist. Brainlab planning software (Brainlab, Munich, Germany) was used for image fusion and either biopsy planning, or intraoperative navigation in case of open tumor resection. [^18^ F] GE180 and FET uptake at the exact localization of the acquired tissue specimen were retrospectively measured by fusing the intraoperative CT or intraoperatively acquired navigation points with the PET images using a Hermes workstation (Hermes Medical Solutions, Stockholm, Sweden).

**Supplementary Fig. 2 TSPO promotor methylation across the entire TSPO gene locus and across GBM methylation subtypes**. Overview of the methylation distribution per probe covering the entire *TSPO* gene locus (n = 15) in TCGA-GBM and TCGA-LGG gliomas **(a)**. Further analyses of *TSPO* mRNA expression (RPKM) and methylation (beta value) in TCGA-GBM and TCGA-LGG *in silico* data sets **(b-d)**. *TSPO* expression was analyzed based on reported GBM methylation subtypes (RTK I: 5 IDH-wt; RTK II: 19 IDH-wt; MES: 14 IDH-wt). There were no significant *TSPO* expression difference between the GBM methylation subtypes (Post hoc Games Howell test) **(b)**. Analysis of TSPO methylation at probe cg00343092 based on GBM methylation subtypes showed a significantly higher *TSPO* methylation in RTK II compared to MES gliomas (Post hoc Games Howell, *p = 0.044). Nevertheless, most beta values were below the 0.5 threshold **(c)**. Spearman rho correlation of matched values does not show an inverse correlation between *TSPO* methylation (probe cg00343092) and *TSPO* mRNA expression in any of the different GBM methylation subtypes **(d)**. Significances are displayed as follows: p > 0.05 = n.s., p < 0.05 = *, p < 0.01 = **, p < 0.001 = ***. CpG: 5’-C-phosphate-G-3’, IDH: isocitrate dehydrogenase, IDH-wt: IDH-wildtype, IDH-mut: IDH-mutant, GBM: glioblastoma, LGG: low-grade glioma, MES: mesenchymal methylation pattern, RPKM: reads per kilobase per million, RTK I: receptor thyrosine kinase I methylation pattern, RTK II: receptor thyrosine kinase II methylation pattern, TCGA: The Cancer Genome Atlas.

**Supplementary Fig. 3 TSPO antibody validation showed a specific staining pattern with no unspecific binding**. Western blot analysis with an anti-TSPO [EPR 5384] and a polyclonal control in two transient TSPO-knockdown (+) glioma cell models (U87 + U251MG) vs. a scrRNA control (-) showing a signal decrease in the TSPO-knockdown sample (+) and only one band at the expected TSPO height (18 kDa) when using anti-TSPO [EPR 5384]. Another polyclonal anti-TSPO antibody showed unspecific staining pattern and was not used further **(a)**. Western blot signal of an antibody epitope blocking experiment in two scrRNA-transfected glioma cell models (U87 + U251MG) vanishes completely when the antibody epitope binding site is blocked **(b)**. Western blot of anti-TSPO [EPR 5384] in 4 GBM lysates demonstrates specific binding patterns in all samples **(c)**. TSPO-IHC shows a very strong TSPO labeling in a TSPO-wildtype control while two TSPO-knockout C20 microglia cell models (B11 + D9) had no detectable TSPO staining **(d)**. Antibody epitope blocking experiment: TSPO-IHC of in the infiltration zone of an anaplastic astrocytoma shows no antibody binding when epitope binding site is blocked, **(e)**. GBM: glioblastoma, IHC: immunohistochemistry, L: ladder, scrRNA: scrambled RNA, siRNA: small interfering RNA pool.

**Supplementary Fig. 4 TSPO-/FET-PET and MRI enrichment of a TSPO-low and a TSPO-high IDH-wildtype glioblastoma**. TSPO-PET overview images of a TSPO-PET low GBM case (GBM_20) **(a)** and a TSPO-PET high GBM case (GBM_10) **(b)**. Corresponding FET-PET overview images of a TSPO-low GBM case **(c)** and a TSPO-high GBM case **(d)**. Corresponding MRI overview images of a TSPO-low GBM case **(e)** and a TSPO-high GBM case **(f)**. Corresponding full H&E staining (400x) of a TSPO-low GBM case **(g)** and a TSPO-high GBM case **(h)**. Corresponding full TSPO-IHC (400x) of a TSPO-low GBM case **(i)** and a TSPO-high GBM case **(j)**. FET: F-18-fluorethyltyrosin, GBM: glioblastoma, MRI: magnetic resonance imaging, PET: positron-emission tomography.

**Supplementary Fig. 5 TSPO group split criteria for RNA sequencing analysis of glioblastoma patients**. Normalized *TSPO* mRNA counts (RNA-Seq, DeSeq normalized counts) correlate with TSPO protein expression (IHC, %TSPO Area) **(a)**. IDH-wt GBMs were grouped together by median split (DeSeq normalized counts cutoff: 92.90) in a *TSPO*-low (9 TSPO LOW) and a *TSPO*-high (9 TSPO HIGH) group for analysis of differentially expressed genes **(b)**. GBM: glioblastoma, IDH: isocitrate dehydrogenase, IDH-wt: IDH-wildtype, IHC: immunohistochemistry, RNA-Seq: RNA sequencing.

**Supplementary Fig. 6 High TSPO expression in glioblastoma indicates the prognostic unfavorable mesenchymal subtype**. Enrichment of mesenchymal signature genes [72] was found in the *TSPO*-high group (marked red) and not in the *TSPO*-low group (marked blue cases) **(a)**. When splitting the *TSPO*-high group further into a TSPO HIGH II and TSPO HIGH III cluster, significant higher *TSPO* expression levels were found in the TSPO HIGH III cluster **(b)**. Spearman rho gene-to gene correlation in patients with IDH-wt GBM (TSPO study cohort, TPMs) showed significant associations between *TSPO* and *STAT3* (r = 0.804, ***p < 0.001), *TSPO* and *OSM* (r = 0.678, **p = 0.002), *TSPO* and *CD44* (r = 0.782, ***p < 0.001), and *TSPO* and *OSMR* (r = 0.502, *p = 0.034) **(c)**. *TSPO* single-cell mRNA expression levels (scRNA-Seq) displayed across reported cell type clusters (malignant cells, macrophages, oligodendrocytes, T cells). TSPO mRNA expression was observed in all these cell types **(d)**. Significances are displayed as follows: p > 0.05 = n.s., p < 0.05 = *, p < 0.01 = **, p < 0.001 = ***. CD44: cluster of differentiation 44, GBM: glioblastoma, IDH: isocitrate dehydrogenase, IDH-wt: IDH-wildtype, IHC: immunohistochemistry, OSM: oncostatin M, OSMR: oncostatin M receptor, scRNA-Seq: single-cell RNA sequencing, STAT3: signal transducer and activator of transcription 3, TPMs: transcripts per million.

**Supplementary Fig. 7 Full H&E and IF images of the GBM cases from Fig. 7e**. Representative images of H&E staining and OPAL multiplex immune fluorescence staining (400x) showed a higher TSPO/CD68-positive cell portion in the TSPO HIGH III (GBM_25) case in comparison to TSPO HIGH II (GBM_11 II) and TSPO LOW I (GBM_17) cases. CD68: cluster of differentiation 68/ macrosialin, DAPI: 4′,6-diamidino-2-phenylindole.

**Supplementary Fig. 8 ETS1/2 expression in non-neoplastic and glioma tissue and correlation to TSPO expression**. *ETS1* and *ETS2* mRNA expression (TCGA-GBM/LGG, TPM by UALCAN query) displayed across reported GBM sample types (Normal Tissues: 5, Primary Tumors: 156) **(a)** and reported LGG histological subtypes (Astrocytoma: 194, Oligoastrocytoma: 130, Oligodendroglioma: 191) **(b)**. Substantial *ETS1/2* mRNA expression levels were observed in GBM and low-grade gliomas, with *ETS1* upregulation and *ETS2* downregulation in GBM in comparison to non-neoplastic brain tissue (p < 0.05). *In silico* spearman rho gene-to gene correlation in 152 GBM samples (TCGA-GBM by cBioportal, RNA Seq V2 RSEM) showed a weak association between *TSPO* and ETS2 (r = 0.301, ***p < 0.001) **(c)**, and no association between *TSPO* and *ETS1* (data not shown). Spearman rho gene-to gene correlation in 18 patients with IDH-wt GBM (our TSPO study cohort, TPMs) also showed a significant association between *TSPO* and *ETS2* (r = 0.668, **p = 0.002) **(d)**. Significances are displayed as follows: p > 0.05 = n.s., p < 0.05 = *, p < 0.01 = **, p < 0.001 = ***. ETS1: ETS Proto-Oncogene 1 transcripton factor, ETS2: ETS Proto-Oncogene 2 transcription factor, GBM: glioblastoma, LGG: low-grade glioma, TCGA: The Cancer Genome Atlas, TPMs: transcripts per million, UALCAN: University of Alabama at Birmingham.

**Supplementary Material 9**: Supplementary Table 1. Overview of TSPO promotor CpG island location (GRCh38, chr22: 43,151,314-43,152,163) with transcription factor binding sites, direct bisulfite PCR methylation and qPCR results per tested sample.

**Supplementary Material 10**: Supplementary Table 2. IDH-wt WHO grade 4 glioma study collective.

**Supplementary Material 11:** Supplementary Table 3. Molecularly analyzed IDH-wt WHO grade 4 glioma study collective.

**Supplementary Material 12**: Supplementary Table 4. Primer and PCR conditions for bisulfite PCR methylation analysis.

**Supplementary Material 13**: Supplementary Table 5. Opal Multiplex IF staining parameter.

## Data Availability

Fastq.files and raw count tables of RNA sequencing data were deposited in GEO (GEO access number: GSE230453). All other data generated or analyzed during this study are included in this published article (and its supplementary information files). Raw data are available from the corresponding author on reasonable request.
