# Supplementary figures and images for: Translocator protein (18kDA) (TSPO) marks mesenchymal glioblastoma cell populations characterized by elevated numbers of tumor-associated macrophages
Source: Acta Neuropathol Commun. 2023 Sep 11;11:147. doi: 10.1186/s40478-023-01651-5 (PMC10496331; doi:10.1186/s40478-023-01651-5)

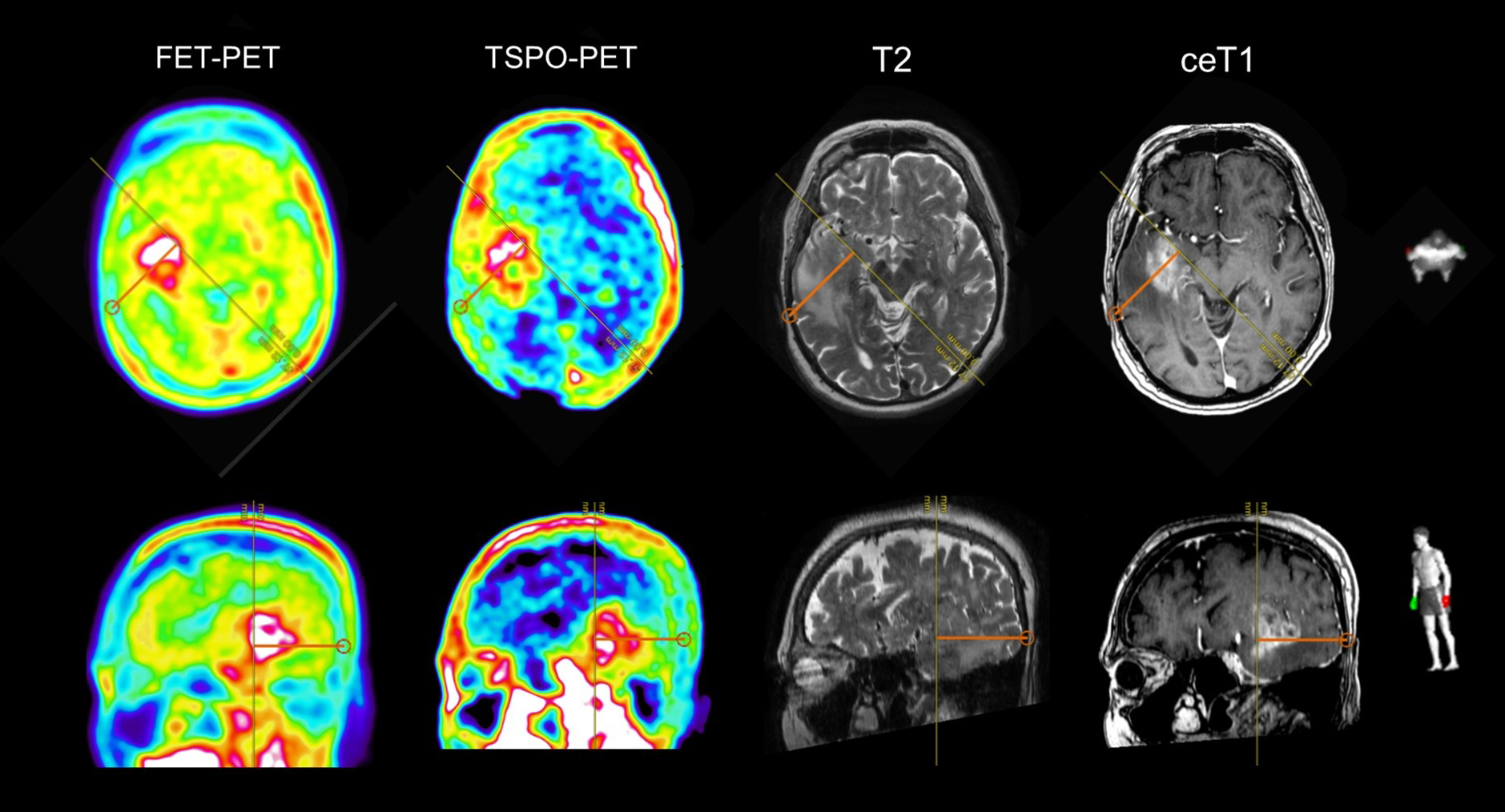

Supplement: Supplementary file 1 — Supplementary Fig. 1 Representative image fusion example generated with Brainlab planning software for the FOR2858 TSPO-PET imaging study. All patients received contrast-enhanced MRI, [18 F]GE180-PET and [18 F]FET-PET within a maximum of 18 and a median of 3 days before the operation. MRI included gadolinium enhanced T1- (1 mm slices) and T2‐weighted scans (2 mm slices). For [18 F]GE180-PET, approximately 180 MBq [18 F]GE180 were injected intravenously and summation scans 60–80 min post injection were used for image analysis. For [18 F]FET-PET, approximately 180 MBq [18 F]FET were injected and 40 min post injection summation images were analyzed as described previously [68]. Areas of interest were defined in an interdisciplinary exchange between the attending neurosurgeon and nuclear medicine specialist. Brainlab planning software (Brainlab, Munich, Germany) was used for image fusion and either biopsy planning, or intraoperative navigation in case of open tumor resection. [18 F] GE180 and FET uptake at the exact localization of the acquired tissue specimen were retrospectively measured by fusing the intraoperative CT or intraoperatively acquired navigation points with the PET images using a Hermes workstation (Hermes Medical Solutions, Stockholm, Sweden). [file 40478_2023_1651_MOESM1_ESM.jpg]

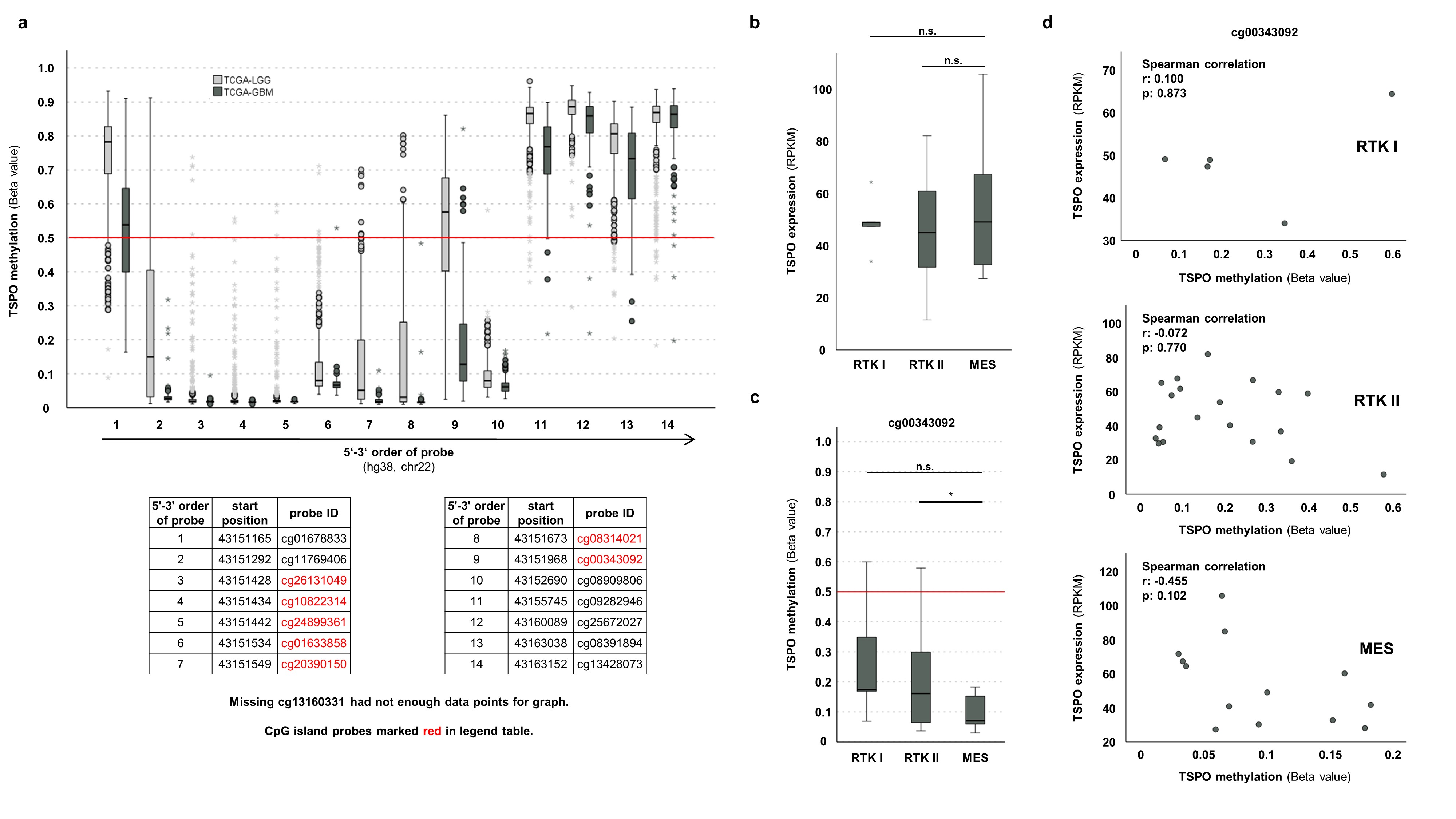

Supplement: Supplementary file 2 — Supplementary Fig. 2 TSPO promotor methylation across the entire TSPO gene locus and across GBM methylation subtypes. Overview of the methylation distribution per probe covering the entire TSPO gene locus (n = 15) in TCGA-GBM and TCGA-LGG gliomas (a). Further analyses of TSPO mRNA expression (RPKM) and methylation (beta value) in TCGA-GBM and TCGA-LGG in silico data sets (b-d). TSPO expression was analyzed based on reported GBM methylation subtypes (RTK I: 5 IDH-wt; RTK II: 19 IDH-wt; MES: 14 IDH-wt). There were no significant TSPO expression difference between the GBM methylation subtypes (Post hoc Games Howell test) (b). Analysis of TSPO methylation at probe cg00343092 based on GBM methylation subtypes showed a significantly higher TSPO methylation in RTK II compared to MES gliomas (Post hoc Games Howell, *p = 0.044). Nevertheless, most beta values were below the 0.5 threshold (c). Spearman rho correlation of matched values does not show an inverse correlation between TSPO methylation (probe cg00343092) and TSPO mRNA expression in any of the different GBM methylation subtypes (d). Significances are displayed as follows: p > 0.05 = n.s., p < 0.05 = *, p < 0.01 = **, p < 0.001 = ***. CpG: 5’-C-phosphate-G-3’, IDH: isocitrate dehydrogenase, IDH-wt: IDH-wildtype, IDH-mut: IDH-mutant, GBM: glioblastoma, LGG: low-grade glioma, MES: mesenchymal methylation pattern, RPKM: reads per kilobase per million, RTK I: receptor thyrosine kinase I methylation pattern, RTK II: receptor thyrosine kinase II methylation pattern, TCGA: The Cancer Genome Atlas. [file 40478_2023_1651_MOESM2_ESM.jpg]

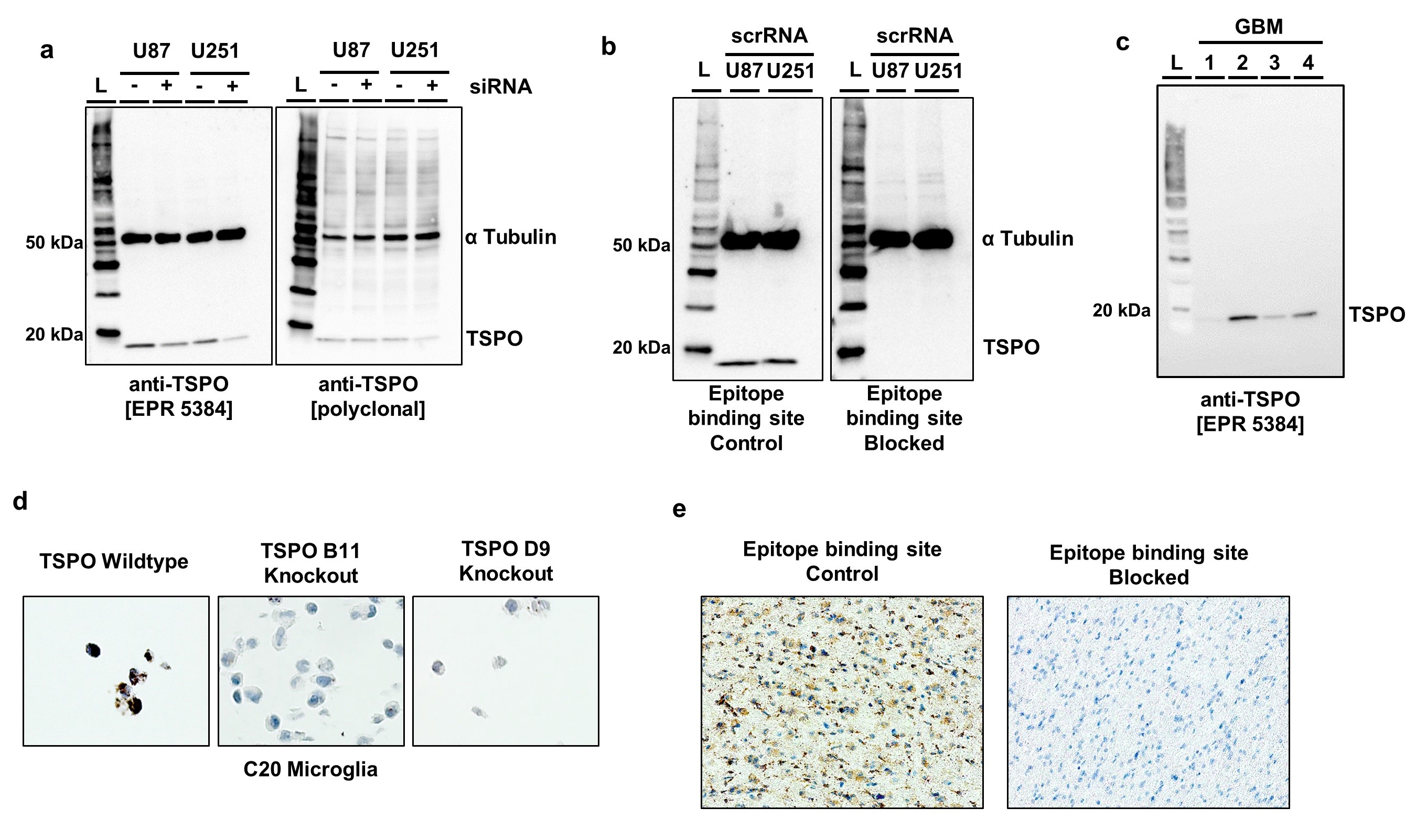

Supplement: Supplementary file 3 — Supplementary Fig. 3 TSPO antibody validation showed a specific staining pattern with no unspecific binding. Western blot analysis with an anti-TSPO [EPR 5384] and a polyclonal control in two transient TSPO-knockdown (+) glioma cell models (U87 + U251MG) vs. a scrRNA control (-) showing a signal decrease in the TSPO-knockdown sample (+) and only one band at the expected TSPO height (18 kDa) when using anti-TSPO [EPR 5384]. Another polyclonal anti-TSPO antibody showed unspecific staining pattern and was not used further (a). Western blot signal of an antibody epitope blocking experiment in two scrRNA-transfected glioma cell models (U87 + U251MG) vanishes completely when the antibody epitope binding site is blocked (b). Western blot of anti-TSPO [EPR 5384] in 4 GBM lysates demonstrates specific binding patterns in all samples (c). TSPO-IHC shows a very strong TSPO labeling in a TSPO-wildtype control while two TSPO-knockout C20 microglia cell models (B11 + D9) had no detectable TSPO staining (d). Antibody epitope blocking experiment: TSPO-IHC of in the infiltration zone of an anaplastic astrocytoma shows no antibody binding when epitope binding site is blocked, (e). GBM: glioblastoma, IHC: immunohistochemistry, L: ladder, scrRNA: scrambled RNA, siRNA: small interfering RNA pool. [file 40478_2023_1651_MOESM3_ESM.jpg]

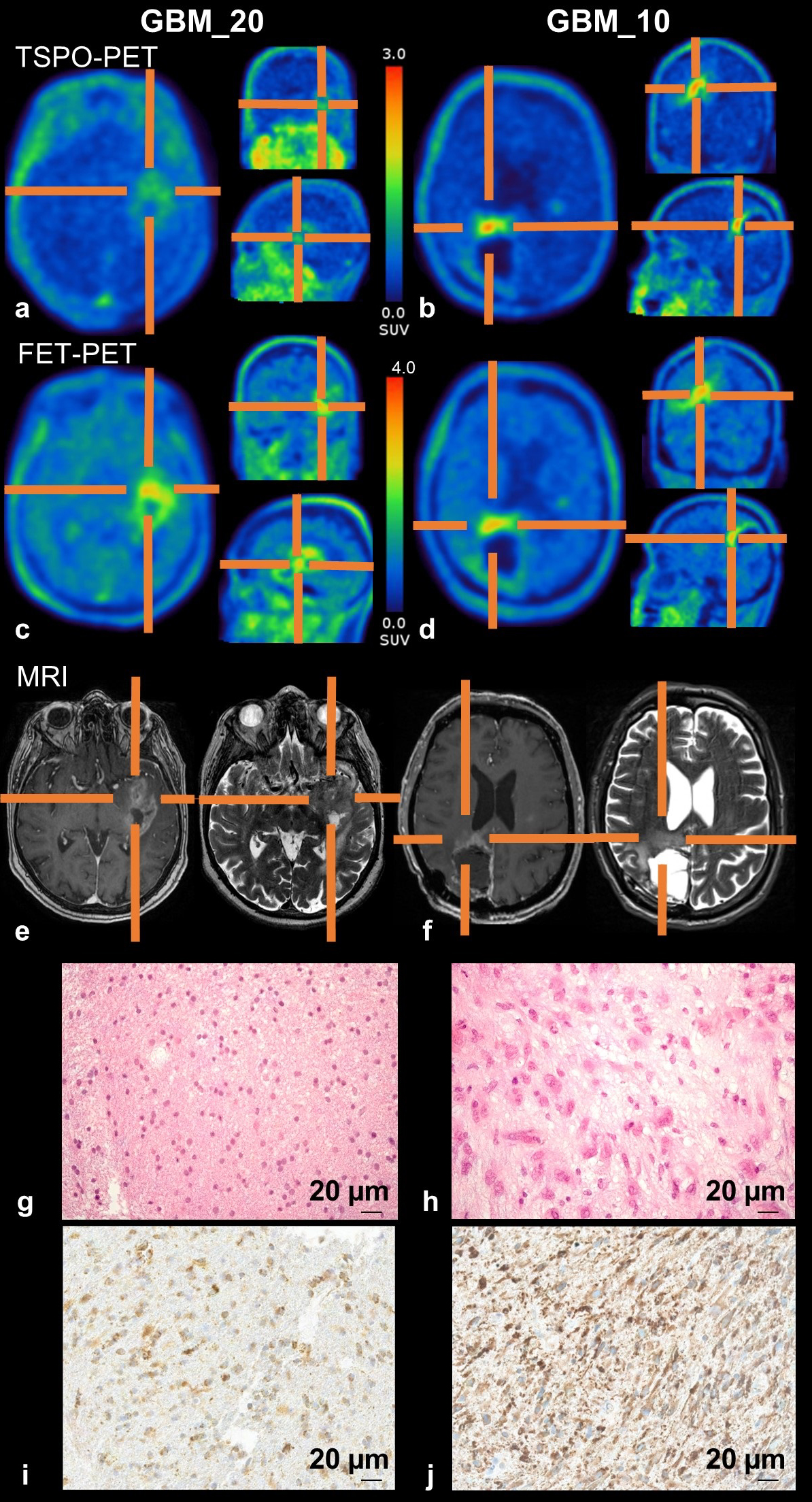

Supplement: Supplementary file 4 — Supplementary Fig. 4 TSPO-/FET-PET and MRI enrichment of a TSPO-low and a TSPO-high IDH-wildtype glioblastoma. TSPO-PET overview images of a TSPO-PET low GBM case (GBM_20) (a) and a TSPO-PET high GBM case (GBM_10) (b). Corresponding FET-PET overview images of a TSPO-low GBM case (c) and a TSPO-high GBM case (d). Corresponding MRI overview images of a TSPO-low GBM case (e) and a TSPO-high GBM case (f). Corresponding full H&E staining (400x) of a TSPO-low GBM case (g) and a TSPO-high GBM case (h). Corresponding full TSPO-IHC (400x) of a TSPO-low GBM case (i) and a TSPO-high GBM case (j). FET: F-18-fluorethyltyrosin, GBM: glioblastoma, MRI: magnetic resonance imaging, PET: positron-emission tomography. [file 40478_2023_1651_MOESM4_ESM.jpg]

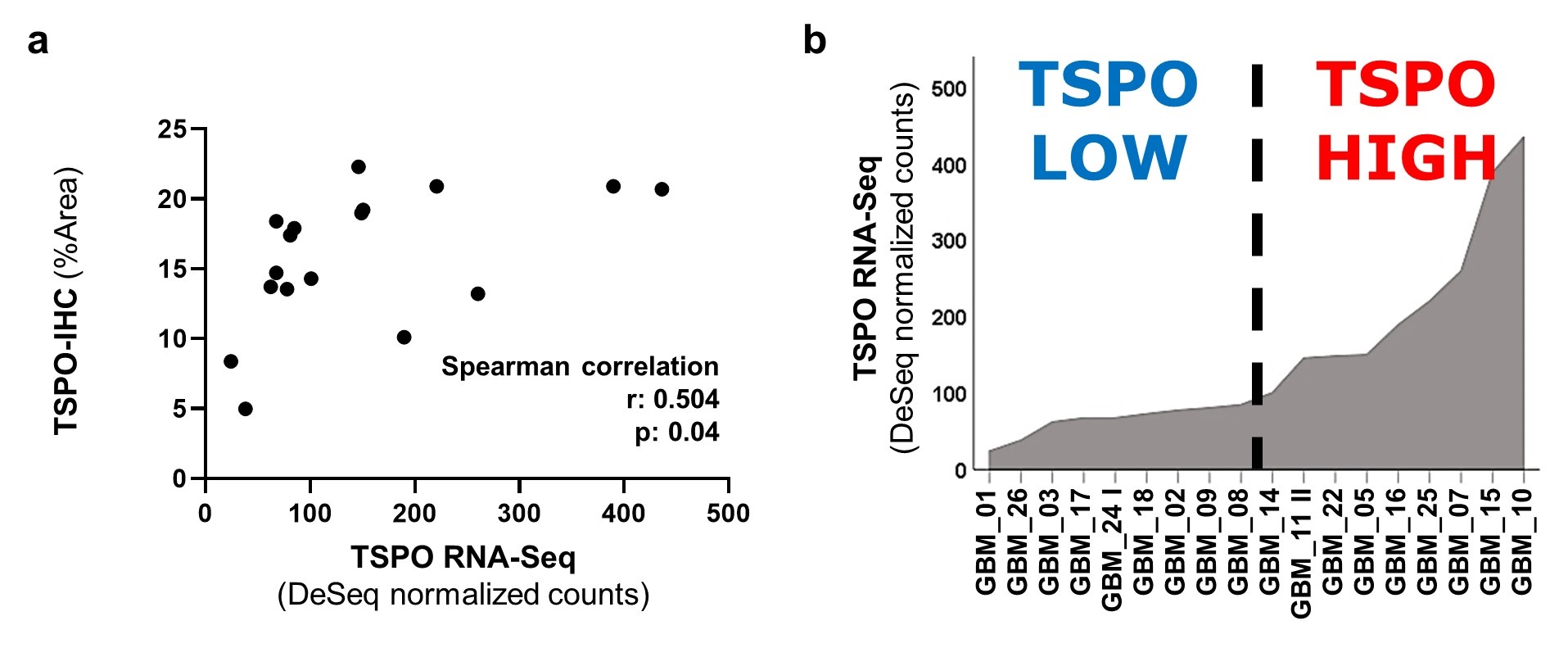

Supplement: Supplementary file 5 — Supplementary Fig. 5 TSPO group split criteria for RNA sequencing analysis of glioblastoma patients. Normalized TSPO mRNA counts (RNA-Seq, DeSeq normalized counts) correlate with TSPO protein expression (IHC, %TSPO Area) (a). IDH-wt GBMs were grouped together by median split (DeSeq normalized counts cutoff: 92.90) in a TSPO-low (9 TSPO LOW) and a TSPO-high (9 TSPO HIGH) group for analysis of differentially expressed genes (b). GBM: glioblastoma, IDH: isocitrate dehydrogenase, IDH-wt: IDH-wildtype, IHC: immunohistochemistry, RNA-Seq: RNA sequencing. [file 40478_2023_1651_MOESM5_ESM.jpg]

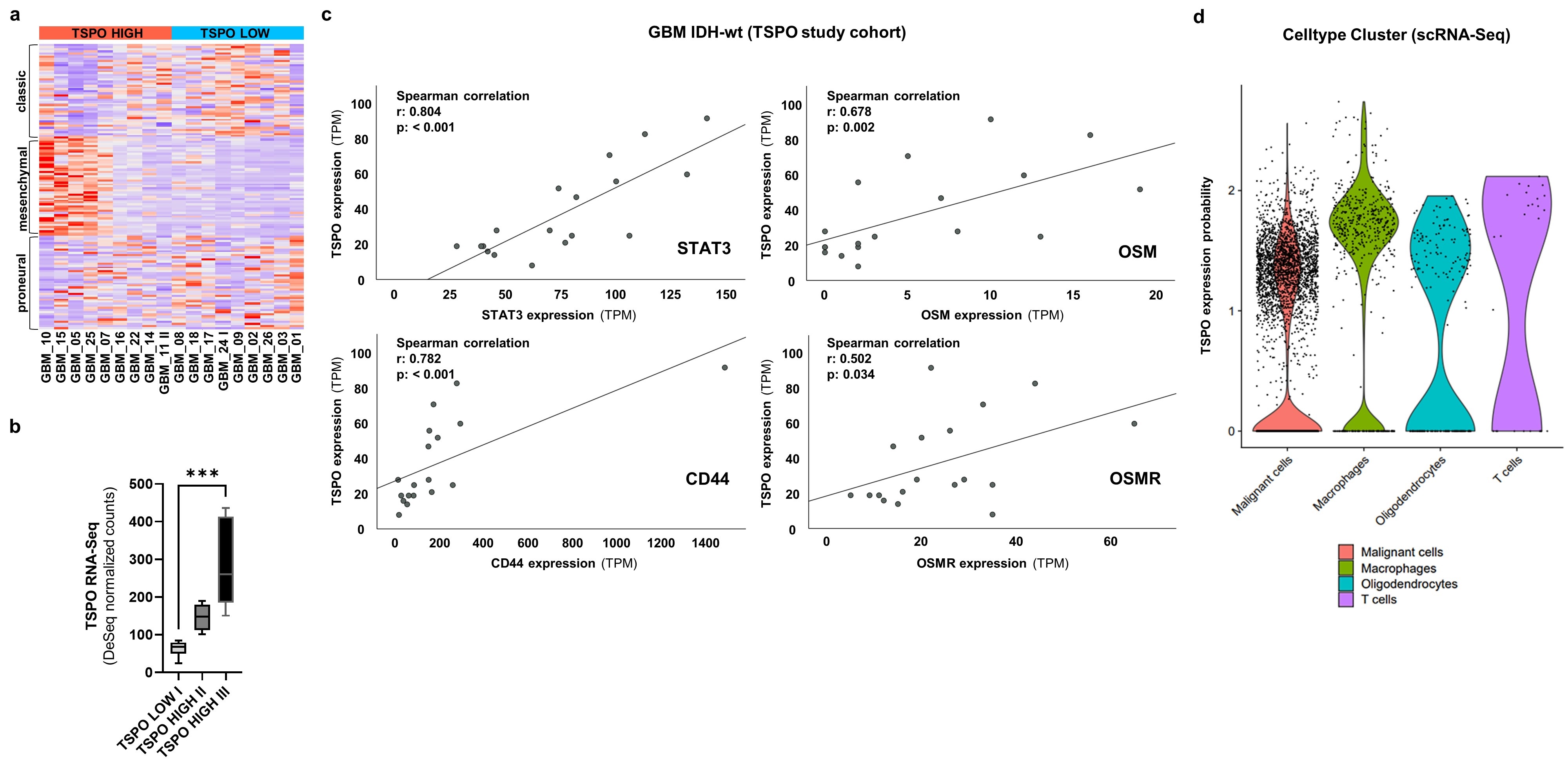

Supplement: Supplementary file 6 — Supplementary Fig. 6 High TSPO expression in glioblastoma indicates the prognostic unfavorable mesenchymal subtype. Enrichment of mesenchymal signature genes [72] was found in the TSPO-high group (marked red) and not in the TSPO-low group (marked blue cases) (a). When splitting the TSPO-high group further into a TSPO HIGH II and TSPO HIGH III cluster, significant higher TSPO expression levels were found in the TSPO HIGH III cluster (b). Spearman rho gene-to gene correlation in patients with IDH-wt GBM (TSPO study cohort, TPMs) showed significant associations between TSPO and STAT3 (r = 0.804, ***p < 0.001), TSPO and OSM (r = 0.678, **p = 0.002), TSPO and CD44 (r = 0.782, ***p < 0.001), and TSPO and OSMR (r = 0.502, *p = 0.034) (c). TSPO single-cell mRNA expression levels (scRNA-Seq) displayed across reported cell type clusters (malignant cells, macrophages, oligodendrocytes, T cells). TSPO mRNA expression was observed in all these cell types (d). Significances are displayed as follows: p > 0.05 = n.s., p < 0.05 = *, p < 0.01 = **, p < 0.001 = ***. CD44: cluster of differentiation 44, GBM: glioblastoma, IDH: isocitrate dehydrogenase, IDH-wt: IDH-wildtype, IHC: immunohistochemistry, OSM: oncostatin M, OSMR: oncostatin M receptor, scRNA-Seq: single-cell RNA sequencing, STAT3: signal transducer and activator of transcription 3, TPMs: transcripts per million. [file 40478_2023_1651_MOESM6_ESM.jpg]

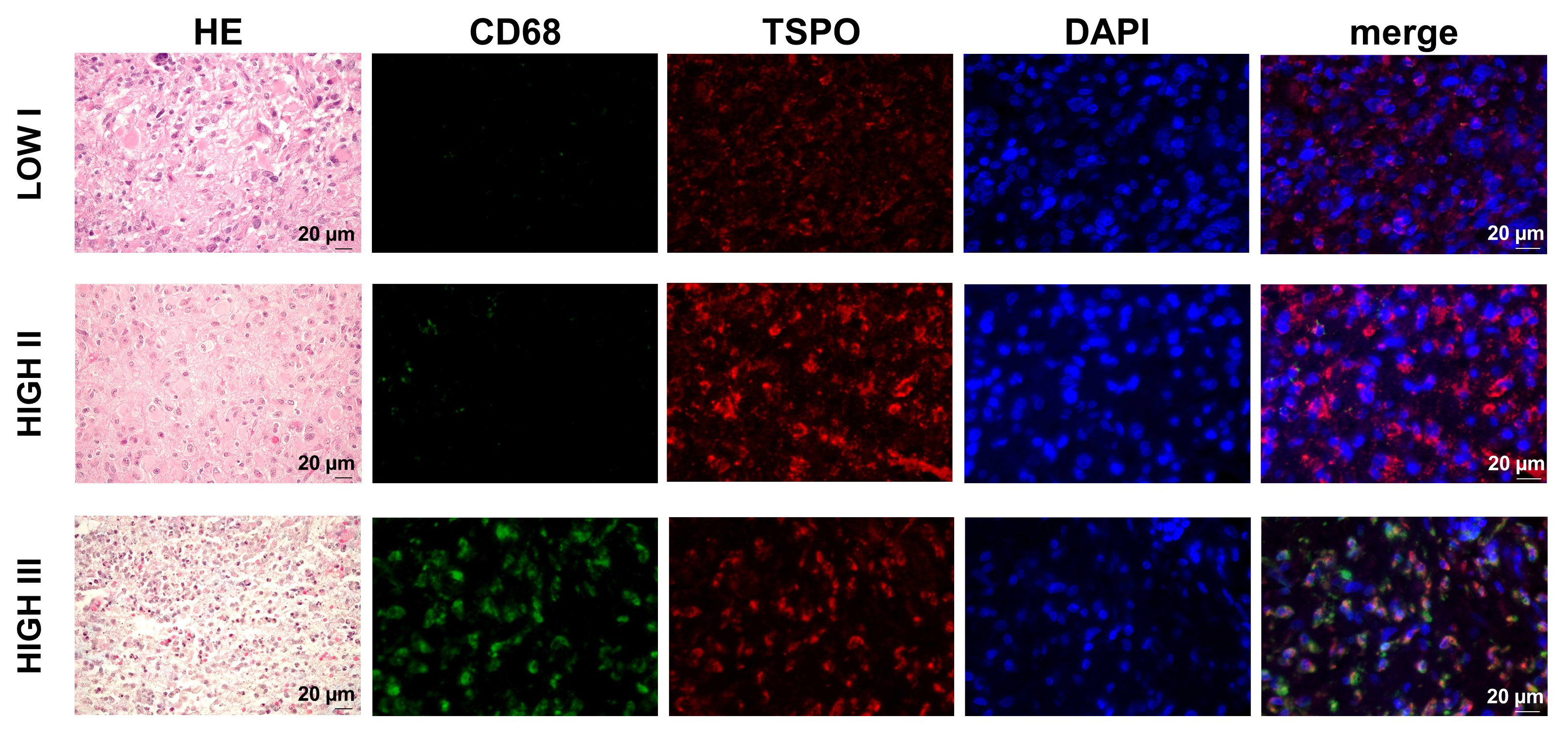

Supplement: Supplementary file 7 — Supplementary Fig. 7 Full H&E and IF images of the GBM cases from Fig. 7e. Representative images of H&E staining and OPAL multiplex immune fluorescence staining (400x) showed a higher TSPO/CD68-positive cell portion in the TSPO HIGH III (GBM_25) case in comparison to TSPO HIGH II (GBM_11 II) and TSPO LOW I (GBM_17) cases. CD68: cluster of differentiation 68/ macrosialin, DAPI: 4′,6-diamidino-2-phenylindole. [file 40478_2023_1651_MOESM7_ESM.jpg]

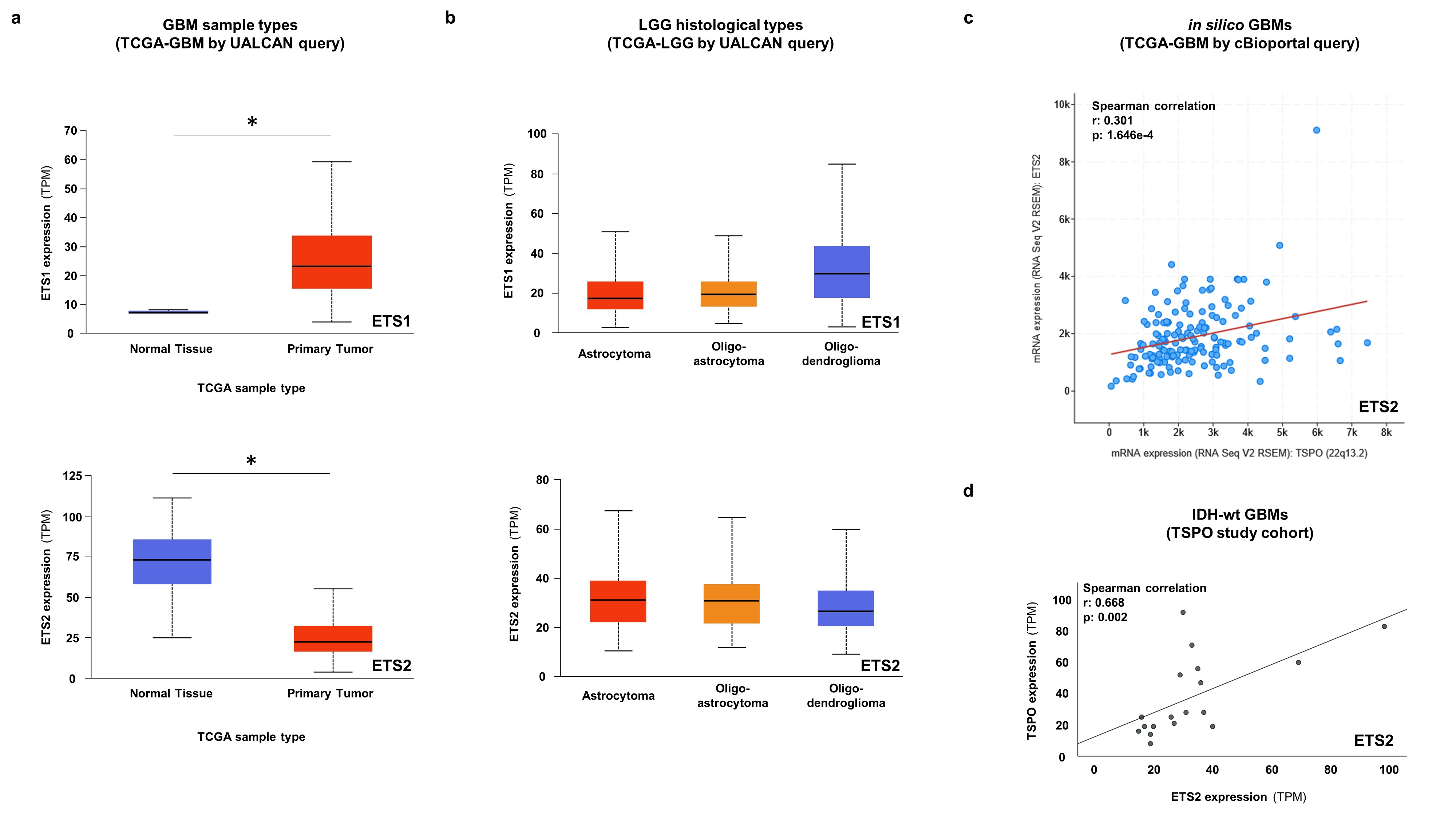

Supplement: Supplementary file 8 — Supplementary Fig. 8 ETS1/2 expression in non-neoplastic and glioma tissue and correlation to TSPO expression. ETS1 and ETS2 mRNA expression (TCGA-GBM/LGG, TPM by UALCAN query) displayed across reported GBM sample types (Normal Tissues: 5, Primary Tumors: 156) (a) and reported LGG histological subtypes (Astrocytoma: 194, Oligoastrocytoma: 130, Oligodendroglioma: 191) (b). Substantial ETS1/2 mRNA expression levels were observed in GBM and low-grade gliomas, with ETS1 upregulation and ETS2 downregulation in GBM in comparison to non-neoplastic brain tissue (p < 0.05). In silico spearman rho gene-to gene correlation in 152 GBM samples (TCGA-GBM by cBioportal, RNA Seq V2 RSEM) showed a weak association between TSPO and ETS2 (r = 0.301, ***p < 0.001) (c), and no association between TSPO and ETS1 (data not shown). Spearman rho gene-to gene correlation in 18 patients with IDH-wt GBM (our TSPO study cohort, TPMs) also showed a significant association between TSPO and ETS2 (r = 0.668, **p = 0.002) (d). Significances are displayed as follows: p > 0.05 = n.s., p < 0.05 = *, p < 0.01 = **, p < 0.001 = ***. ETS1: ETS Proto-Oncogene 1 transcripton factor, ETS2: ETS Proto-Oncogene 2 transcription factor, GBM: glioblastoma, LGG: low-grade glioma, TCGA: The Cancer Genome Atlas, TPMs: transcripts per million, UALCAN: University of Alabama at Birmingham. [file 40478_2023_1651_MOESM8_ESM.jpg]
